# Illustrated type catalogue of *Amphidromus* Albers, 1850 in the Natural History Museum, London, and descriptions of two new species

**DOI:** 10.3897/zookeys.492.8641

**Published:** 2015-03-30

**Authors:** Chirasak Sutcharit, Jonathan Ablett, Piyoros Tongkerd, Fred Naggs, Somsak Panha

**Affiliations:** 1 Animal Systematics Research Unit, Department of Biology, Faculty of Science, Chulalongkorn University, Bangkok 10330, Thailand; 2 Division of Higher Invertebrates, Natural History Museums, London, SW7 5BD, United Kingdom

**Keywords:** Tree snails, systematics, molluscs, type specimen, Southeast Asia, NHM, taxonomy

## Abstract

The collection of the Southeast Asian tree snail genus *Amphidromus* Albers, 1850 at the Natural History Museum, London includes more than 100 lots of type specimens representing 85 name-bearing types, 9 paratypes and 6 paralectotypes, and one *nomen nudum*. Lectotypes are here designated for *Amphidromus cambojiensis*, *Amphidromus perakensis globosus*, *Amphidromus columellaris gloriosa*, *Amphidromus maculiferus inflata*, *Amphidromus lepidus*, *Amphidromus sinistralis lutea*, *Amphidromus moniliferus*, *Amphidromus maculiferus obscura*, *Amphidromus sinistralis rosea* and *Amphidromus sinensi vicaria*. In addition, the missing types of A.A. Gould were discovered and their type status is discussed. A complete catalogue of these types, including colour photographs is provided for the first time. After examining these type specimens, two new *Amphidromus* species, Amphidromus (Syndromus) globonevilli Sutcharit & Panha, **sp. n.** and Amphidromus (Syndromus) principalis Sutcharit & Panha, **sp. n.** were recognized and are described herein.

## Introduction

*Amphidromus* Albers, 1850 is a genus of tree dwelling snails; the members of this genus are distributed in the region from Assam in India throughout Indochina, the southern of the Philippines, Indonesia (east of Weber’s line) with a single species occurring in the Northern Territory of Australia (Pilsbry 1900, Solem 1959, 1983, Laidlaw and Solem 1961, Sutcharit and Panha 2006). This diverse genus of large snails with colorful shells has long been known to malacologists. The first revision of *Amphidromus* by Fulton (1896a) arranged nominal species into 19 species groups, and included descriptions of new species with illustrations. Pilsbry’s revision (1900) provided more complete descriptions and redescriptions and figured species, some for the first time, becoming the standard identification guide for the group. Laidlaw and Solem (1961) gathered and documented further information on previously recognised species and provided a list of all species-group names applied to *Amphidromus*. The most significant issue of Laidlaw and Solem (1961) was the recording of the primary type specimens, the institution where they were deposited and registration number for all the species within the entire genus. More than 300 nominal species-group names have been applied to this genus (Richardson 1985), but only 75 were recognized as distinct species in Laidlaw and Solem (1961), since then an additional 16 species have been describied and validated (see Solem 1983, Dharma 1993, 2007, Panha 1996, Lehmann and Maassen 2004, Severns 2006, Sutcharit and Panha 2006b, 2011, Chan and Tan 2010, Cilia 2013). This indicates that *Amphidromus* are morphologically variable, especially in shell colour, which has led to an over-description of some taxa, and species recognition based solely on published descriptions and figures are being difficult. Therefore, type specimens are the ultimate reference point for species identification, and represent an international standard providing the basis of nomenclatural stability when following the International Code of Zoological Nomenclature (ICZN). Comparison with the primary type specimens will minimise this difficulty, at least within the constraints of morphological taxonomy.

The Natural History Museum in London (hereafter the NHM), formerly the British Museum (Natural History), is one of the oldest and largest museum collections with mollusc specimens acquired from many varied sources and collectors (Dance 1986). Two collections that contain important type material of the genus *Amphidromus* are those of Hugh Cuming (containing 27 type specimens of *Amphidromus* described by L. Pfeiffer and L. Reeve) and Hugh Fulton (included 60 type specimens of *Amphidromus*). These two collections were deposited at the NHM and form the largest collection of primary type specimens of *Amphidromus*, being comprised of 87 taxa (~one-fourth of the currently known *Amphidromus* taxa). Until now, many of these types have not been figured or adequately figured (Laidlaw and Solem 1961). The second largest collection of *Amphidromus* type material is in the Senckenberg Forschungsinstitut und Naturmuseum in Frankfurt (51 taxa), where all the specimen lots have been catalogued and illustrated (Zilch 1953). Thirty-three type lots of *Amphidromus* are housed in the National Museum of Natural History, Smithsonian Institution the remaining type lots are distributed amongst other museums. However, the primary types of 57 taxa had not previously been traced (Laidlaw and Solem 1961). Some of these ‘missing’ lots have subsequently been traced such as those located at the National Museum of Wales, Cardiff (Wood and Gallichan 2008).

Recent research on *Amphidromus* systematics including detailed morphological studies of reproductive anatomy and molecular phylogenetics (Sutcharit et al. 2007) needs to be integrated with a critical assessment of type material. This will allow for the correct application of nomenclature and the recognition of suitable voucher specimens that can act as surrogates of type specimens for DNA and additional morphological work, since historical species were often described based solely on shells. The aim of this paper is to evaluate the type status of *Amphidromus* type specimens in the NHM collections and to figure specimens and designate lectotypes in acordence with ICZN (1999: Art. 74) guidelines. Evaluating species as biological entities is largely outside of the scope of this study. However, examination of these type collections, revealed two *Amphidromus* species that we consider to be new and these are described herein.

## Materials and methods

**Collections:** The primary type specimens (i.e. holotype, lectotype and syntype/syntypes) along with the paratype(s) and paralectotype(s) of *Amphidromus* described from the early 19^th^ century until 2013 and deposited at the NHM were examined. Those specimens that were confirmed as forming part of the type series of species, where a unique type had not been designated, were considered to be syntype lots. In cases where a holotype was not explicitly designated but where in the original publication the species name was clearly based on an individual shell, these were taken to be the holotype fixed by monotypy. Lectotypes mentioned in this catalogue have been designated by Laidlaw and Solem (1961), unless otherwise stated, and conform to the ICZN guidelines (1999).

**Figure 1.**
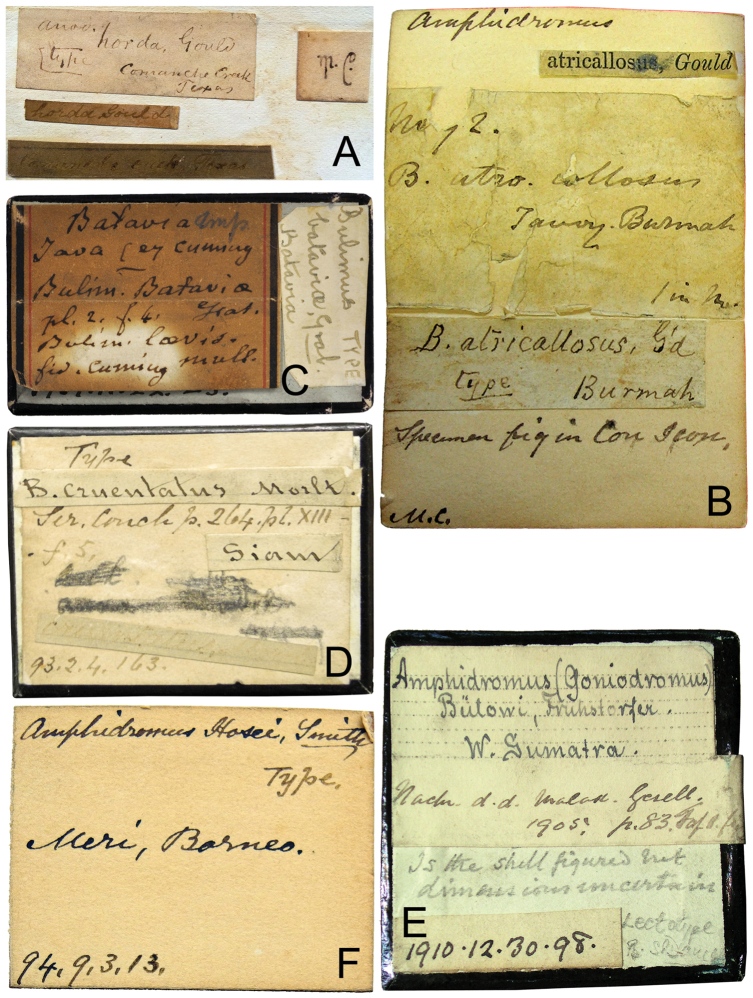
Original labels of the type specimens. **A** Evidence of the original labels of Gould’s type specimens in the H. Cuming collection. Label of *Anodonta horda* Gould, 1855, lectotype (NHMUK 196465) designated by Johnson (1964: 88). The original label marked with “Type” does not frequently occur in H. Cuming’s collection, which suggests that the specimen was received from Gould **B** Label of *Amphidromus atricallosus* (Gould, 1843), the printed label attached on the top is typical of the way that Reeve used to indicate the specimen examined and figured in the Conchologica Iconica **C** Label of *Amphidromus bataviae* (Grateloup, 1840) **D** Label of *Amphidromus bulowi* Fruhstorfer, 1905 **E** Label of *Amphidromus cruentatus* (Morelet, 1875) **F** Label of *Amphidromus hosei* Smith, 1895.

From the published list of Gould’s type specimens, Johnson (1964) presumed that some of the unlocated types were probably to be found in the NHM. Although, most of A. Gould’s types can be found in the Museum of Comparative Zoology, Harvard University, there was a record that Gould presented the specimens of some species that he had described to Hugh Cuming (Johnson 1964). Among Gould’s types that were unequivocally recognized in the NHM, the original labels are obviously marked with “Type” and their locality is congruent with the recorded type locality. For example, Johnson (1964: 88) certainly accepted the type specimen of “*Anodonta horda* Gould, 1855” was in the H. Cuming collection and designated a specimen (NHMUK registration no. 196465) as the lectotype (Fig. 1A). Such evidence is, therefore, taken into account in order to distinguish Gould’s type specimens.

**Figure 2.**
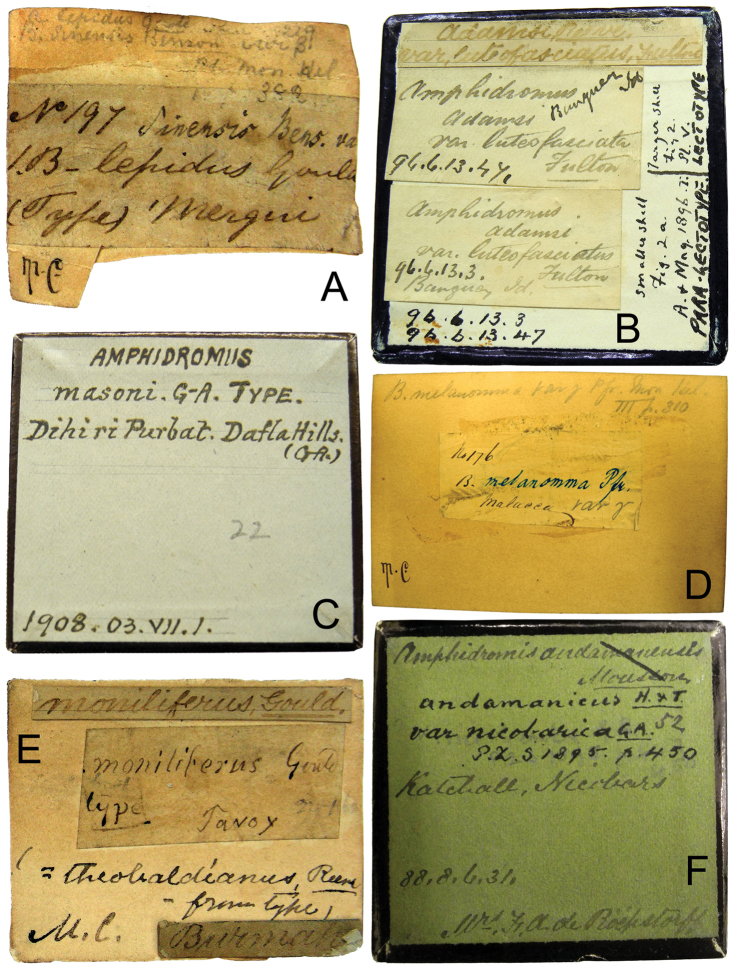
Original labels of the type specimens. **A** Label of *Amphidromus lepidus* (Gould, 1856), with Pfeiffer’s handwritten “*sinensis* Bens. var.” **B** Bottom of a box with *Amphidromus adamsii luteofasciata* type specimens. The two larger glued labels are Fulton’s original handwritten ones. On the right side, the vertical lines indicate an unambiguously designated lectotype in Laidlaw and Solem (1961) with reference to Fulton’s (1896) original figures **C** Label of *Amphidromus masoni* (Godwin-Austen, 1876), with Godwin-Austen’s handwritten the species and locality names **D** Label of *Amphidromus melanomma* (Pfeiffer, 1852), with Pfeiffer’s handwritten of the specie name in blue ink **E** Label of *Amphidromus moniliferus* (Gould, 1846), the name “*theobaldianus*, Reeve – from type” was subsequently added up later **F** Label of *Amphidromus andamanicus nicobarica* Godwin-Austen, 1895.

This illustrated catalogue provides the shell measurements and photographs of the name-bearing types. All specimens considered as forming part of the type series were photographed in the standard position, apertural and abapertural views. Additional views were also photographed for the taxa that have unique shell characters. The original labels were photographed and checked with the original description (Figs 1, 2). Measurements of any holotype and lectotype material were taken in mm with digital calipers. Those taxa where the primary type is housed in a different institution to the NHM, but where paratypes or paralectotypes are kept in the NHM, are also included in this illustrated catalogue.

**Structure of the illustrated catalogue:** The taxa in this illustrated type catalogue were checked against the original publications and are listed as given in the original description regardless of termination or incorrect original spelling, and the authorship(s) and date. Additional comments, such as the print date, availability of the name or corrected subsequent spelling, are provided in square brackets. The synonymy tabulation and the usage of each taxon name are provided in Pilsbry (1900), Laidlaw and Solem (1961) and Richardson (1985). Only the original combination of the taxon name with reference to pages, plate and/or figures are mentioned. The type locality is given verbatim as stated in the original publication. If possible, the modern name and/or regional names of the type locality are provided in square brackets. If any incongruence between the published type locality and that given on the original label occurred, this is mentioned in the comments under the remarks of those taxa. Under the type materials, primary type specimens with the NMH registration number (registered specimens are cited as NHMUK), the measurements of shell height (H) and shell width (W), and the figures are given. In addition, if the paratypes or paralectotypes of that taxa are available then the respective registration number, number of specimens with a dextral (D) or sinistral (S) coiling direction, and figures of a representative specimen are given. If necessary, remarks are given on the status of type specimens, authorships, availability of name, notes on the type locality, and other necessary comments. Full bibliographic references are provided at the end of this paper.

**Institutional abbreviation:** Abbreviations of the museum collections used the lists of taxa and species descriptions are listed as follows:

CUMZ Chulalongkorn University, Museum of Zoology, Bangkok, Thailand

MCZ Museum of Comparative Zoology, Harvard University, Cambridge, UK

MNHN Muséum National ďHistoire Naturelle, Paris, France

MZB Museum Zoologicum Bogoriense, Indonesia

NHMUK Natural History Museum, London, UK

RMNH National Museum of Natural History, Leiden, Netherlands

SMF Forschungsinstitut und Naturmuseum Senckenberg, Frankfurt a.m., Germany

UMZC University Museum of Zoology Cambridge, Cambridge, UK

ZMA Zoological Museum of Amsterdam, Amsterdam, Netherlands

## Results

There are 210 type specimens representing 100 available names within the genus *Amphidromus* in the NHM collections. Only one species name “*globosa* Nevill, 1878” is considered as an unavailable nominal taxon (ICZN 1999: Art. 12). Among these available names, the NHM retained 85% of the name-bearing types exclusively as 10 holotypes, 70 lectotypes and five lots of syntype material. Of the 10 holotype lots, a lot of “*nicobarica* Godwin-Austen, 1895” was recently discovered in the general collections and recognized as the holotype (fixed by monotypy). The five syntypes are “*gracilior* Fulton, 1896”, “*melanomma* Pfeiffer, 1852”, “*robustus* Fulton, 1896”, “*rubiginosa* Fulton, 1896” and “*theobaldianus* Benson, 1857”. Among the 70 lectotype lots, ten lots were recently designated from the original type series of W. Collinge “*globosus* Fulton, 1903” and type series of H. Fulton as “*gloriosa* Fulton, 1896”, “*inflata* Fulton, 1896”, “*lutea* Fulton, 1896”, “*obscura* Fulton, 1896”, “*rosea* Fulton, 1896” and “*vicaria* Fulton, 1896”. The three long unrecognized type series of H. Cuming “*cambojiensis* Reeve, 1860”, “*lepidus* Gould, 1856” and “*moniliferus* Gould, 1846” are discovered. They are acknowledged as lectotypes to clarify their type status and promote the stability of the taxon name. The history and type evidences are summarized under each taxon.

The remaining 15% are paratypes and paralectotypes, whose name-bearing types had been designated and housed elsewhere. The original type series of six nominal taxa (“*atricallosus* Gould, 1843”, “*begini* Morlet, 1886”, “*romaensis* Rolle, 1903”, “*roseotincta* Möllendorff, 1894”, “*singalangensis* Rolle, 1908” and “*ventrosulus* Möllendorff, 1900”) are recently recognized taxa in the NHM, and are considered as paralectotypes. The other nine nominal taxa of “*abbasi* Chan and Tan, 2010”, “*albulus* Sutcharit and Panha, 2006”, “*babiensis* Laidlaw, 1954”, “*banksi* Butot, 1955”, “*classiarius* Sutcharit and Panha, 2006”, “*dextrochlorus* Sutcharit and Panha, 2006”, “*iunior* Cilia, 2013”, “*rottinensis* Chan and Tan, 2010” and “*simalurensis* Laidlaw, 1954” have only the paratypes available at the NHM.

### Alphabetical list of the taxa

#### Amphidromus abbasi

Taxon classificationAnimaliaStylommatophoraCamaenidae

Chan & Tan, 2010

Amphidromus abbasi Chan & Tan, 2008: 7, 8, fig. 1. [*nomen nudum*, ICZN 1999: Arts 8.6 and 11.1].
Amphidromus abbasi Chan & Tan, 2010: 246, fig. 1a–c.

##### Type locality.

Approximately 1.2 km from coast, Laggaliru, Southwest Sumba, Indonesia.

##### Type material.

**Figure 3.**
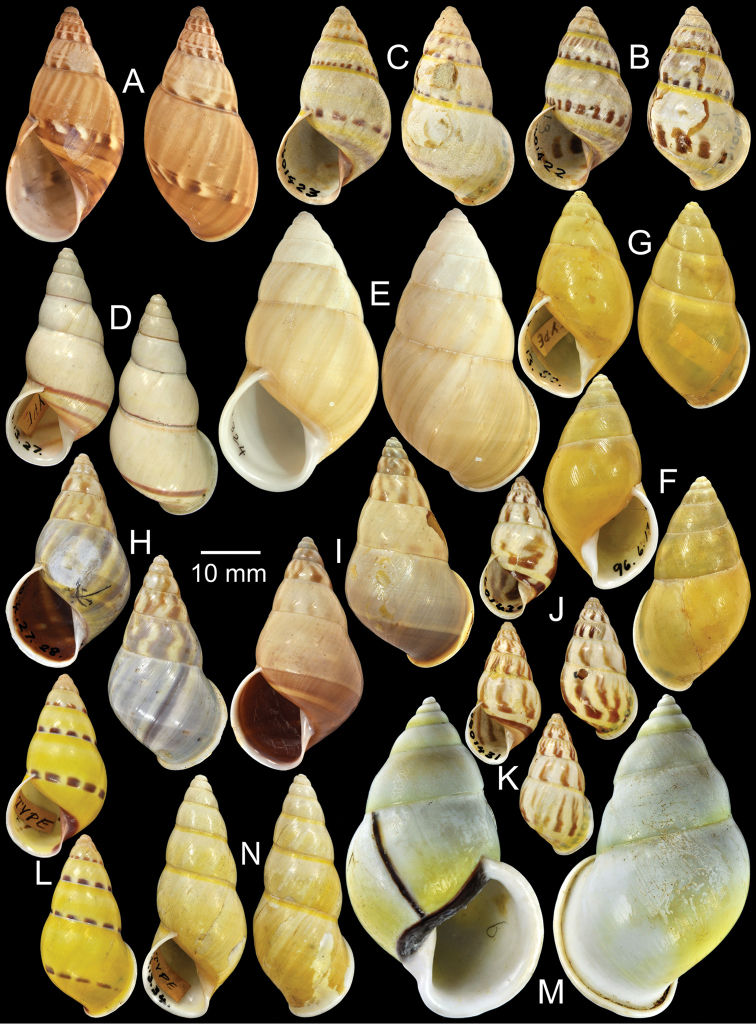
Type specimens of *Amphidromus* spp. **A** Paratype of *Amphidromus abbasi* **B–C** *Amphidromus adamsii*, **B** lectotype and **C** paralectotype **D** Lectotype of *Amphidromus suspectus albolabiatus* **E** Paratype of *Amphidromus inversus albulus* **F–G** *Amphidromus alticola*, **F** lectotype and **G** paralectotype **H–I** *Amphidromus angulatus*, **H** lectotype and **I** paralectotype **J–K** *Amphidromus areolatus*, **J** lectotype and **K** paralectotype **L** Lectotype of *Amphidromus adamsii articulata* **M** Paralectotype of *Amphidromus atricallosus* **N** Lectotype of *Amphidromus adamsii aureocincta*.

Holotype MZB-Gastropoda 14.232, paratypes NHMUK 20080623 (2S, Fig. 3A).

##### Remarks.

Chan and Tan (2008) described “*abbasi*” in the Occasional Molluscan Papers which does not fulfill the ICZN guidelines and could not be made available (ICZN 1999: Arts 8.6 and 11.1). However, “*abbasi*” was later published correctly (ICZN 1999: Art. 8) and made available in Chan and Tan (2010).

The authors stated that three specimens were housed in the NHM under this paratype lot, but only two are registered in the NHM collections (Chan and Tan 2010).

#### Amphidromus adamsii

Taxon classificationAnimaliaStylommatophoraCamaenidae

(Reeve, 1848)

Bulimus adamsii Reeve, 1848: *Bulimus* plate 13, species 73, fig. 73a–d.

##### Type locality.

Eastern Coast of Borneo (on a tall tree in an islet between Banguey and Balambangan).

##### Type material.

Lectotype NHMUK 19601422 (Fig. 3B; H=30.0 mm, W=16.8 mm), paralectotypes NHMUK 19601423 (2S, Fig. 3C).

##### Remarks.

There is a variation in the spelling of the species name, of which “*adamsi*” is considered as an incorrect subsequent spelling. References of the subsequent use of the incorrect spelling have been compiled in Laidlaw and Solem (1961: 597). The original and correct spelling is “*adamsii*”.

#### Amphidromus suspectus albolabiata

Taxon classificationAnimaliaStylommatophoraCamaenidae

Fulton, 1896

Amphidromus suspectus var. albolabiatus Fulton, 1896a: 79, pl. 6, fig. 9.

##### Type locality.

Timor.

##### Type material.

Lectotype NHMUK 1896.6.3.27 (Fig. 3D; H=36.9 mm, W=17.8 mm).

#### Amphidromus inversus albulus

Taxon classificationAnimaliaStylommatophoraCamaenidae

Sutcharit & Panha, 2006

Amphidromus inversus albulus Sutcharit & Panha, 2006a: 80–82, figs 2–4.

##### Type locality.

Kapas Island (Pulau Kapas), Marang, Terengganu, peninsular Malaysia.

##### Type material.

Holotype CUMZ 2323, paratypes CUMZ 2299 (3D + 8S), CUMZ 2300 (5D + 17S), CUMZ 2324 (4D + 1S), CUMZ 2327 (14D + 20S), NHMUK 20050160 (1D + 1S, Fig. 3E), SMF 327982 (1D + 1S).

#### Amphidromus alticola

Taxon classificationAnimaliaStylommatophoraCamaenidae

Fulton, 1896

Amphidromus alticola (Boettger, MSS.), Fulton 1896a: 70, pl. 6, fig. 5, 5a.

##### Type locality.

Java.

##### Type material.

Lectotype NHMUK 1896.6.13.49 (Fig. 3F; H=34.7 mm, W=18.8 mm), paralectotype NHMUK 1896.6.13.50 (1D, Fig. 3G).

##### Remarks.

Fulton wrote “Boettger, MSS.” after the species name, but it appears that there was no description by O. Boettger. The taxon is, therefore, attributed solely to Fulton.

#### Amphidromus angulatus

Taxon classificationAnimaliaStylommatophoraCamaenidae

Fulton, 1896

Amphidromus angulatus Fulton, 1896a: 84, 85, pl. 6, fig. 3.

##### Type locality.

Sarawak.

##### Type material.

Lectotype NHMUK 1889.4.27.28 (Fig. 3H; H=35.1 mm, W=19.2 mm), paralectotypes NHMUK 1889.4.27.29 (2S, Fig. 3I).

#### Amphidromus areolatus

Taxon classificationAnimaliaStylommatophoraCamaenidae

(Pfeiffer, 1861)

Bulimus areolatus Pfeiffer, 1861: 194.

##### Type locality.

Siam [Thailand].

##### Type material.

Lectotype NHMUK 19601430 (Fig. 3J; H=22.5 mm, W=11.9 mm), paralectotype NHMUK 19601431 (1S, Fig. 3K).

#### Amphidromus adamsii articulata

Taxon classificationAnimaliaStylommatophoraCamaenidae

Fulton, 1896

Amphidromus adamsi var. articulata Fulton, 1896a: 82, pl. 5, fig. 7.

##### Type locality.

Banguey Island [Sabah, Malaysia].

##### Type material.

Lectotype NHMUK 1896.6.13.2 (Fig. 3L; H=31.0 mm, W=16.0 mm).

#### Amphidromus atricallosus

Taxon classificationAnimaliaStylommatophoraCamaenidae

(Gould, 1843)

Bulimus atricallosus Gould, 1843: 140.
Bulimus atricallosus —Gould 1844: 457, pl. 24 fig. 3.

##### Type locality.

Tavoy, British Burma [Dawei, Tanintharyi Region, Myanmar].

##### Type material.

Lectotype (designated by Johnson 1964: 44), MCZ 169050, paralectotype NHMUK 20110203 (Figs 1B, 3M; H=54.1 mm, W=33.3 mm).

##### Remarks.

Gould (1844: 457) mentioned that two specimens were the basis for the species description, but did not explicitly designate a holotype. Johnson (1964: 44) stated that “figured holotype MCZ 169050”, but this specimen does not match with the original figure, especially in the differing location of the dark varix (Gould 1844: pl. 24, fig. 3). The holotype that Johnson specified seems to be inappropriate, and should be interpreted as a lectotype designation (ICZN 1999: Art. 74.6) to stabilise the name. In addition, the “paratype FMNH 72403” mentioned in Sutcharit and Panha (2006b: 14) is misinterpreted. This specimen from the Laidlaw ex. Fulton collection from the type locality should be considered as a topotype.

The dextral specimen, from the H. Cuming collection and figured in Reeve (1848), has an original label stating “type” and the locality is congruent with the type locality (Fig. 1B). This supports that supposition that the specimen likely came from Gould’s type series and is, therefore, considered as the paralectotype. In addition, Johnson (1964: 88) recognized a sinistral specimen as “paratype MCZ 169051”. However, if this sinistral specimen originated from the original type series, Gould would have most likely mentioned the sinistral specimen in the original description and is in the opionion of the authors unlikely to be type material.

#### Amphidromus aureocincta

Taxon classificationAnimaliaStylommatophoraCamaenidae

Fulton, 1896

Amphidromus adamsi var. aureocincta Fulton, 1896a: 83, 84, pl. 5 fig. 3, 3a.

##### Type locality.

North Borneo.

##### Type material.

Lectotype NHMUK 1896.6.13.34 (Fig. 3N; H=41.0 mm, W=17.2 mm), paralectotypes SMF 7551 (2S).

#### Amphidromus webbi babiensis

Taxon classificationAnimaliaStylommatophoraCamaenidae

Laidlaw, 1954

Amphidromus webbi babiensis Laidlaw, 1954: 76–78, fig. 1.

##### Type locality.

Poeloe Babi Island, Sumatra [Babi Island, Aceh, Indonesia].

##### Type material.

**Figure 4.**
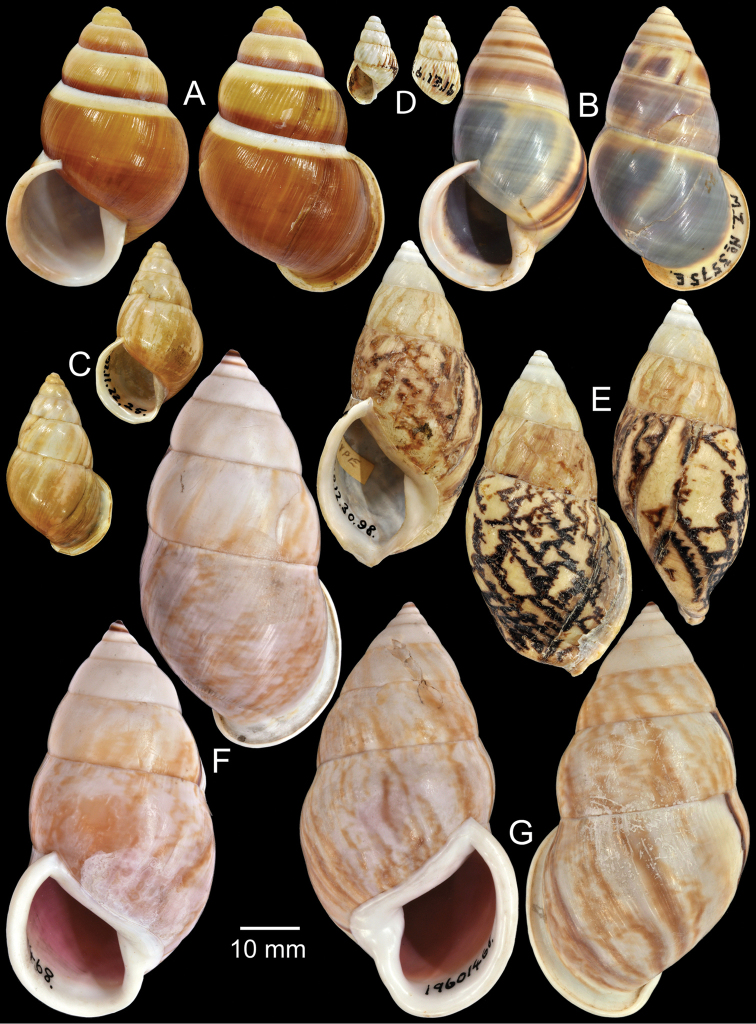
Type specimens of *Amphidromus* spp. **A** Paratype of *Amphidromus webbi babiensis* **B** Paratype of *Amphidromus banksi* **C** Lectotype of *Amphidromus bataviae* **D** Paralectotype of *Amphidromus begini* **E** Lectotype of *Amphidromus bulowi* **F–G** *Amphidromus cambojiensis* **F** lectotype and **G** paralectotype.

Holotype in RMNH, paratype NHMUK 1957.11.18.1 (1S, Fig. 4A).

#### Amphidromus banksi

Taxon classificationAnimaliaStylommatophoraCamaenidae

Butot, 1955

Amphidromus banksi Butot, 1955: 127–129, fig. 29a, pl. 5, figure top left.

##### Type locality.

Mt. Kadam region, Pualu Panaitan, West Java [Pulau Panaitan, Banten, Indonesia].

##### Type material.

Holotype ZMA Moll. 137447, paratypes NHMUK 1957.11.18.3 (1S, Fig. 4B), SMF 153479 (11S).

#### Amphidromus bataviae

Taxon classificationAnimaliaStylommatophoraCamaenidae

(Grateloup, 1840)

Bulimus bataviae Grateloup, 1840b (March): 165.
Partula bataviae Grateloup, 1840a (November): 425, pl. 2, fig. 12.

##### Type locality.

Batavia [Jakarta, Indonesia].

##### Type material.

Lectotype NHMUK 1907.11.22.25 (Figs 1C, 4C; H=30.3 mm, W=17.3 mm).

##### Remarks.

Jean P.S. de Grateloup described “*Bulimus bataviae*” in March 1840. Consecutively, he redescribed this taxon including an illustration in November 1840 as “*Partula bataviae*” (Fig. 1C). However, the previous published name was refered to in the November publication. Therefore, the *Bulimus* name confers the availability, and agrees with Sherborn’s (1922) list which was made available in Grateloup’s March publication (1840b).

#### Amphidromus begini

Taxon classificationAnimaliaStylommatophoraCamaenidae

(Morlet, 1886)

Bulimus begini Morlet, 1886: 74.

##### Type locality.

Plateau de Stang-Trang, Cambodge [Stung Treng Plateau, Cambodia].

##### Type material.

Lectotype (designated by Fischer-Piette 1950: 158) MNHN-IM 2000-1832, paralectotype NHMUK 1896.6.13.16 (1S juvenile, Fig. 4D).

##### Remarks.

The original description does not include an illustration, however Morlet (1889: 177, 178, pl. 6, fig. 4) re-published the description and included illustrations of the species. Fischer-Piette (1950: 158) cited a specimen in the Muséum National ďHistoire Naturelle, Paris collections as the “holotype, 25 mm” which we consider an inadvertent lectotype designation (ICZN 1999: Art. 74.5). The NHM specimen is from the H. Fulton collection ex. Dautzenberg and ex. Morlet and gives “Cambodia” as the collection locality. It is considered to be a paralectotype.

#### Amphidromus bulowi

Taxon classificationAnimaliaStylommatophoraCamaenidae

Fruhstorfer, 1905

Amphidromus bülowi Fruhstorfer, 1905: 83, 84, pl. 1 fig. 2 (lectotype is lower figure).

##### Type locality.

West Sumatra.

##### Type material.

Lectotype NHMUK 1910.12.30.98 (Figs 1E, 4E; H=54.5 mm, W=27.9 mm).

#### Amphidromus cambojiensis

Taxon classificationAnimaliaStylommatophoraCamaenidae

(Reeve, 1860)

Bulimus cambojiensis Reeve, 1860: 204.

##### Type locality.

Cambojia [Cambodia].

##### Type material.

Lectotype (design. n.), NHMUK 19601468/1 (Fig. 4F; H=66.6 mm, W=35.1 mm), paralectotypes NHMUK 19601468/2-3 (1S + 1D, Fig. 4G).

##### Remarks.

*Bulimus cambojiensis* Reeve, 1860 was described from a specimen collected by H. Mouhot. When describing *Bulimus cambojiensis*, Reeve did not designate a unique type. Fulton (1896a) figured this species for the first time, but did not clearly state their syntype status. The specimen that most closely matches the original description (Reeve 1860: 204) and the figure in Fulton (1896a: pl. 7, fig. 7) is designated here as the lectotype to stabilise the name.

Variation in the spelling of the species name is found as “*cambodjensis*” or “*cambogiensis*”, but both are considered as incorrect subsequent spellings (Morelet 1875: 260, Pfeiffer 1877: 23). The correct original spelling “*cambojiensis*” is here highlighted to be maintained as proper usage.

#### Amphidromus chloris

Taxon classificationAnimaliaStylommatophoraCamaenidae

Reeve, 1848

Bulimus chloris Reeve, 1848: *Bulimus* plate 37, species 223, fig. 223.

##### Type locality.

Eastern Islands [probably in the area of Mindanao Islands, Philippines].

##### Type material.

**Figure 5.**
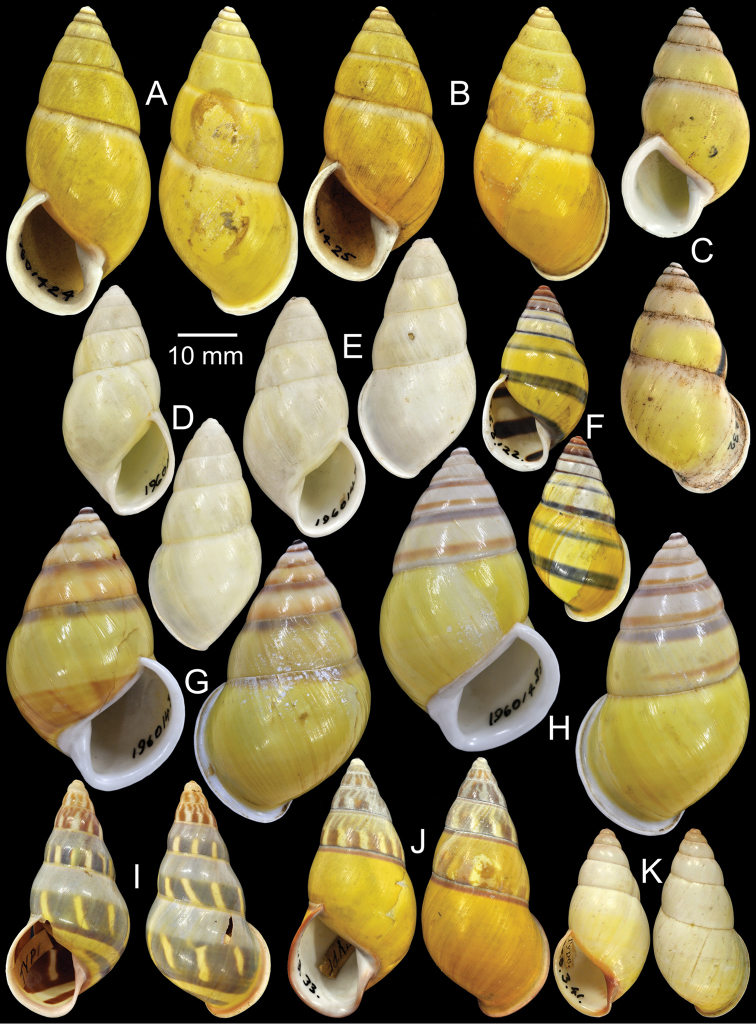
Type specimens of *Amphidromus* spp. **A–B** *Amphidromus chloris* **A** lectotype and **B** paralectotype **C** Paratype of *Amphidromus atricallosus classiarius* **D–E** *Amphidromus cochinchinensis*, **D** lectotype and **E** paralectotype **F** Holotype of *Amphidromus cognatus* **G–H** *Amphidromus comes*, **G** lectotype and **H** paralectotype **I** Lectotype of *Amphidromus pictus concinna* **J** Lectotype of *Amphidromus everetti connectens* **K** Lectotype of *Amphidromus consobrinus*.

Lectotype NHMUK 19601424 (Fig. 5A; H=50.7 mm, W=22.9 mm), paralectotypes NHMUK 19601425 (4S, Fig. 5B), SMF 28065 (2S).

##### Remarks.

The locality on the original label of the type series states the sample was collected from the Philippine Islands. The type locality is, therefore, confined to the Philippines Islands. This is congruent with the known distribution of this species from the Mindanao and Sulu archipelagoes of the Philippines (Bartsch 1917).

#### Amphidromus atricallosus classiarius

Taxon classificationAnimaliaStylommatophoraCamaenidae

Sutcharit & Panha, 2006

Amphidromus atricallosus classiarius Sutcharit & Panha, 2006b: 22, figs 4h, i, 11d–f, 12d, 13d, 14e, f.

##### Type locality.

Koh Tachai, Pangnga, southern Thailand in the Andaman sea.

##### Type material.

Holotype CUMZ 2215, paratypes CUMZ 2011 (10S), 2232 (5S), NHMUK 20050158 (1S, Fig. 5C), SMF 327980 (1S).

#### Amphidromus cochinchinensis

Taxon classificationAnimaliaStylommatophoraCamaenidae

(Pfeiffer, 1857)

Bulimus cochinchinensis Pfeiffer, 1857a [1856]: 331, 332.

##### Type locality.

Cochin China [Southern Vietnam].

##### Type material.

Lectotype NHMUK 19601432 (Fig. 5D; H=38.5 mm, W=19.0 mm), paralectotype NHMUK 19601433 (1D, Fig. 5E).

#### Amphidromus cognatus

Taxon classificationAnimaliaStylommatophoraCamaenidae

Fulton, 1907

Amphidromus cognatus Fulton, 1907: 151, pl. 9, fig. 7.

##### Type locality.

unknown.

##### Type material.

Holotype NHMUK 1907.5.3.122 (Fig. 5F; H=31.0 mm, W=16.5 mm).

##### Remarks.

The type locality was said to be unknown. However, Solem (1983: 154) examined the specimens from precisely known localities, and confined the type locality to be from Port Essington, Cobourg Peninsula, Northern Territory, Australia.

#### Amphidromus comes

Taxon classificationAnimaliaStylommatophoraCamaenidae

(Pfeiffer, 1861)

Bulimus comes Pfeiffer, 1861: 193, 194.

##### Type locality.

Camboja [Cambodia].

##### Type material.

Lectotype NHMUK 19601434 (Fig. 5G; H=46.7 mm, W=28.1 mm), paralectotypes NHMUK 19601435 (2D, Fig. 5H).

#### Amphidromus concinna

Taxon classificationAnimaliaStylommatophoraCamaenidae

Fulton, 1896

Amphidromus pictus var. concinna Fulton, 1896a: 85, pl. 5, fig. 9.

##### Type locality.

Kina Balu, North Borneo [Sabah, Malaysia].

##### Type material.

Lectotype NHMUK 1896.6.13.18 (Fig. 5I; H=35.4 mm, W=17.8 mm).

#### Amphidromus everetti connectens

Taxon classificationAnimaliaStylommatophoraCamaenidae

Fulton, 1896

Amphidromus everetti var. connectens Fulton, 1896a: 87, pl. 5, fig. 17 [= fig. 18 on the plate].

##### Type locality.

North Borneo.

##### Type material.

Lectotype NHMUK 1896.6.13.33 (Fig. 5J; H=43.1 mm, W=20.8 mm).

#### Amphidromus consobrinus

Taxon classificationAnimaliaStylommatophoraCamaenidae

Fulton, 1897

Amphidromus consobrinus Fulton, 1897: 211, 212, pl. 6, fig. 3.

##### Type locality.

South Flores Island [East Nusa Tenggara, Indonesia]; Sumba Island [East Nusa Tenggara, Indonesia].

##### Type material.

Lectotype NHMUK 1897.8.3.41 (Fig. 5K; H=31.9 mm, W=15.5 mm), paralectotype NHMUK 1897.8.3.42 (1S) from South Flores.

##### Remarks.

Fulton stated in the original description that the type series were from two localities. The specimen figured in the original description was designated as the lectotype by Laidlaw and Solem (1961: 611). As a result the type locality of this taxon is restricted to “South Flores Island, East Nusa Tenggara, Indonesia”, the locality of the lectotype.

#### Amphidromus contusus

Taxon classificationAnimaliaStylommatophoraCamaenidae

(Reeve, 1848)

Bulimus contusus Reeve, 1848: *Bulimus* plate 37, species 220, fig. 220.

##### Type locality.

Eastern Islands.

##### Type material.

**Figure 6.**
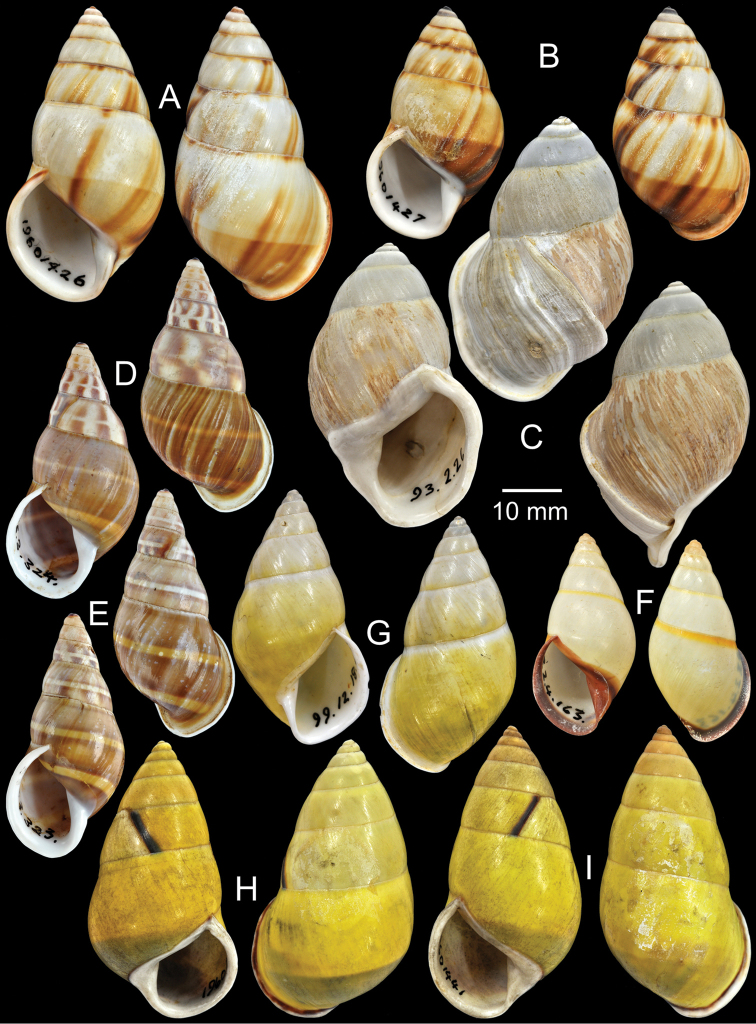
Type specimens of *Amphidromus* spp. **A–B** *Amphidromus contuses* **A** lectotype and **B** paralectotype **C** Holotype of *Amphidromus costifer* **D–E** *Amphidromus contrarius crassa* **D** lectotype and **E** paralectotype **F** Holotype of *Amphidromus cruentatus* **G** Holotype of *Amphidromus dautzenbergi* **H–I** *Amphidromus dohrni* **H** lectotype and **I** paralectotype.

Lectotype NHMUK 19601426 (Fig. 6A; H=49.4 mm, W=25.9 mm), paralectotypes NHMUK 19601427 (3S, Fig. 6B).

#### Amphidromus costifer

Taxon classificationAnimaliaStylommatophoraCamaenidae

Smith, 1893

Amphidromus costifer Smith, 1893: 12 with text fig.

##### Type locality.

Annam [Central Vietnam].

##### Type material.

Holotype NHMUK 1893.2.26.4 (Fig. 6C; H=46.5 mm, W=29.0 mm).

#### Amphidromus crassa

Taxon classificationAnimaliaStylommatophoraCamaenidae

Fulton, 1899

Amphidromus contrarius var. crassa Fulton, 1899a: 213, 215, pl. 11, fig. 8.

##### Type locality.

Timor Island.

##### Type material.

Lectotype NHMUK 1898.12.3.324 (Fig. 6D; H=40.4 mm, W=19.4 mm), paralectotype NHMUK 1898.12.3.323 (1S, Fig. 6E).

#### Amphidromus cruentatus

Taxon classificationAnimaliaStylommatophoraCamaenidae

(Morelet, 1875)

Bulimus cruentatus Morelet, 1875: 264, 265, pl. 13, fig. 5.

##### Type locality.

Cambodje [Cambodia].

##### Type material.

Holotype NHMUK 1893.2.4.163 (Figs 1D, 6F; H=33.4 mm, W=16.5 mm).

#### Amphidromus dautzenbergi

Taxon classificationAnimaliaStylommatophoraCamaenidae

Fulton, 1899

Amphidromus dautzenbergi Fulton, 1899b: 303, fig. 3.

##### Type locality.

Tonkin [Central Vietnam].

##### Type material.

Holotype NHMUK 1899.12.18.38 (Fig. 6G; H=42.9 mm, W=22.6 mm).

#### Amphidromus schomburgki dextrochlorus

Taxon classificationAnimaliaStylommatophoraCamaenidae

Sutcharit & Panha, 2006

Amphidromus schomburgki dextrochlorus Sutcharit & Panha, 2006b: 23–26, figs 4m, 16d–f, 17f.

##### Type locality.

Ban Khok Klang, Tao Ngoi District, Sakonnakhon, northeastern Thailand.

##### Type material.

**Figure 7.**
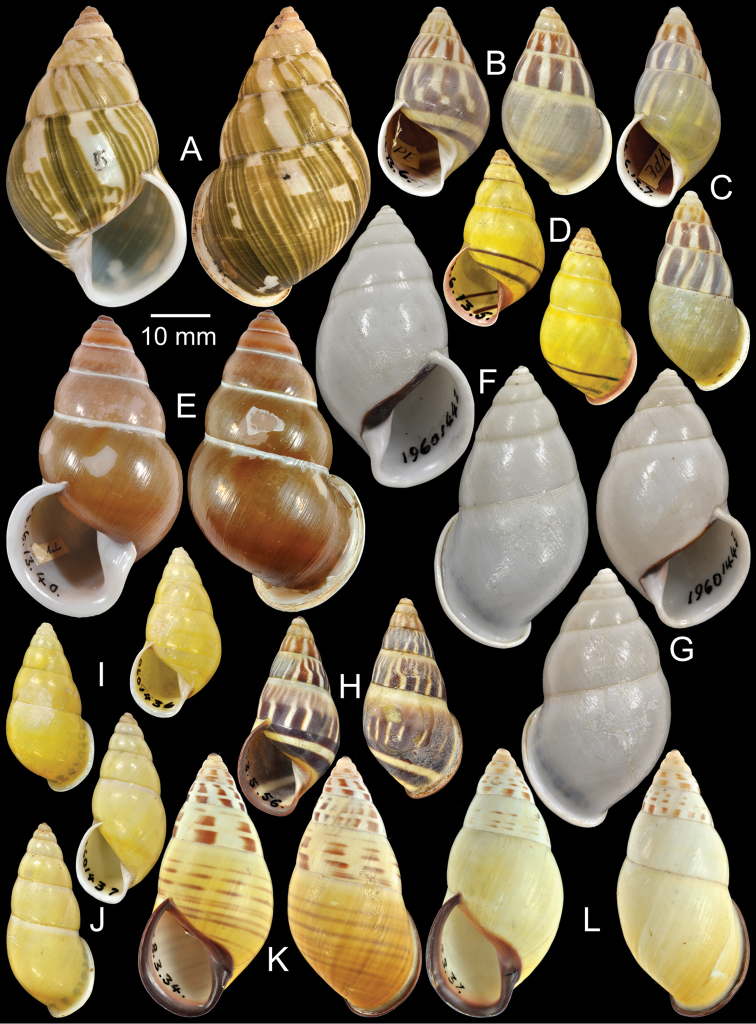
Type specimens of *Amphidromus* spp. **A** Paratype of *Amphidromus schomburgki dextrochlorus* **B–C** *Amphidromus dubius* **B** lectotype and **C** paralectotype **D** Holotype of *Amphidromus adamsii duplocincta* **E** Lectotype of *Amphidromus enganoensis* **F–G** *Amphidromus eques* **F** lectotype and **G** paralectotype **H** Lectotype of *Amphidromus everetti* **I–J** *Amphidromus flavus* **I** lectotype and **J** paralectotype **K–L** *Amphidromus floresianus*, **K** lectotype and **L** paralectotype.

Holotype CUMZ 2296, paratypes CUMZ 2017 (19D), NHMUK 20050149 (1D, Fig. 7A), SMF 327973 (1D).

#### Amphidromus dohrni

Taxon classificationAnimaliaStylommatophoraCamaenidae

(Pfeiffer, 1864)

Bulimus dohrni Pfeiffer, 1864 [1863]: 525.

##### Type locality.

Cochin-China [Southern Vietnam].

##### Type material.

Lectotype NHMUK 19601440 (Fig. 6H; H=46.3 mm, W=24.3 mm), paralectotypes NHMUK 19601441 (1D + 1S, Fig. 6I).

#### Amphidromus dubius

Taxon classificationAnimaliaStylommatophoraCamaenidae

Fulton, 1896

Amphidromus dubius Fulton, 1896a: 86–87, pl. 6, figs 1, 1a.

##### Type locality.

Balabac Island [Palawan, Philippines].

##### Type material.

Lectotype NHMUK 1896.6.13.6 (Fig. 7B; H=31.4 mm, W=17.7 mm), paralectotype NHMUK 1896.6.13.7 (1S, Fig. 7C).

#### Amphidromus adamsii duplocincta

Taxon classificationAnimaliaStylommatophoraCamaenidae

Fulton, 1896

Amphidromus adamsi var. duplocincta Fulton, 1896a: 82, pl. 5, fig. 4.

##### Type locality.

Banguey Island [Sabah, Malaysia].

##### Type material.

Holotype NHMUK 1896.6.13.5 (Fig. 7D; H=29.2 mm, W=15.7 mm).

#### Amphidromus enganoensis

Taxon classificationAnimaliaStylommatophoraCamaenidae

Fulton, 1896

Amphidromus enganoensis Fulton, 1896a: 71, pl. 6, fig. 11.

##### Type locality.

Engano Island, West Sumatra.

##### Type material.

Lectotype NHMUK 1896.6.13.40 (Fig. 7E; H=50.5 mm, W=29.2 mm).

##### Remarks.

The original description was based on more than one specimen and three sets of measurements were given. The unique type was not explicitly designated, and the single specimen that remained in Fulton’s collection could not be implied to be the unique type (ICZN 1999: Art. 74.6). The “holotype” referred to in Laidlaw and Solem (1961) is explicit with a unique indication that constitutes a valid lectotype designation. Therefore, this specimen should be recognized as the lectotype to stabilise the name.

#### Amphidromus eques

Taxon classificationAnimaliaStylommatophoraCamaenidae

(Pfeiffer, 1857)

Bulimus eques Pfeiffer, 1857b: 158.

##### Type locality.

Cochinchina [Southern Vietnam].

##### Type material.

Lectotype NHMUK 19601442 (Fig. 7F; H=47.8 mm, W=26.2 mm), paralectotypes NHMUK 19601443 (2D, Fig. 7G).

#### Amphidromus everetti

Taxon classificationAnimaliaStylommatophoraCamaenidae

Fulton, 1896

Amphidromus everetti Fulton, 1896a: 87.

##### Type locality.

Palawan [Philippines].

##### Type material.

Lectotype NHMUK 1893.3.5.56 (Fig. 7H; H=33.9 mm, W=16.5 mm), paralectotypes SMF 7558 (2S), SMF 7575 (1S), SMF 7663 (1S).

##### Remarks.

The type locality in the original description was given as Palawan. However, the locality on the label of the lectotype is Balabac Island, the southernmost of the Palawan Islands.

#### Amphidromus flavus

Taxon classificationAnimaliaStylommatophoraCamaenidae

(Pfeiffer, 1861)

Bulimus flavus Pfeiffer, 1861: 194.

##### Type locality.

Siam [Thailand].

##### Type material.

Lectotype NHMUK 19601436 (Fig. 7I; H=27.6 mm, W=14.5 mm), paralectotypes NHMUK 19601437 (1S, Fig. 7J).

#### Amphidromus floresianus

Taxon classificationAnimaliaStylommatophoraCamaenidae

Fulton, 1897

Amphidromus floresianus Fulton, 1897: 211, pl. 6, fig. 2.

##### Type locality.

South Flores [Indonesia].

##### Type material.

Lectotype NHMUK 1897.8.3.34 (Fig. 7K; H=44.0 mm, W=21.8 mm), paralectotypes NHMUK 1897.8.3.35–7 (3S, Fig. 7L), SMF 7554 (1S).

#### Amphidromus glaucolarynx

Taxon classificationAnimaliaStylommatophoraCamaenidae

(Dohrn, 1861)

Bulimus glaucolarynx Dohrn, 1861: 207, pl. 26, fig. 7.

##### Type locality.

In regno Siam [Thailand].

##### Type material.

**Figure 8.**
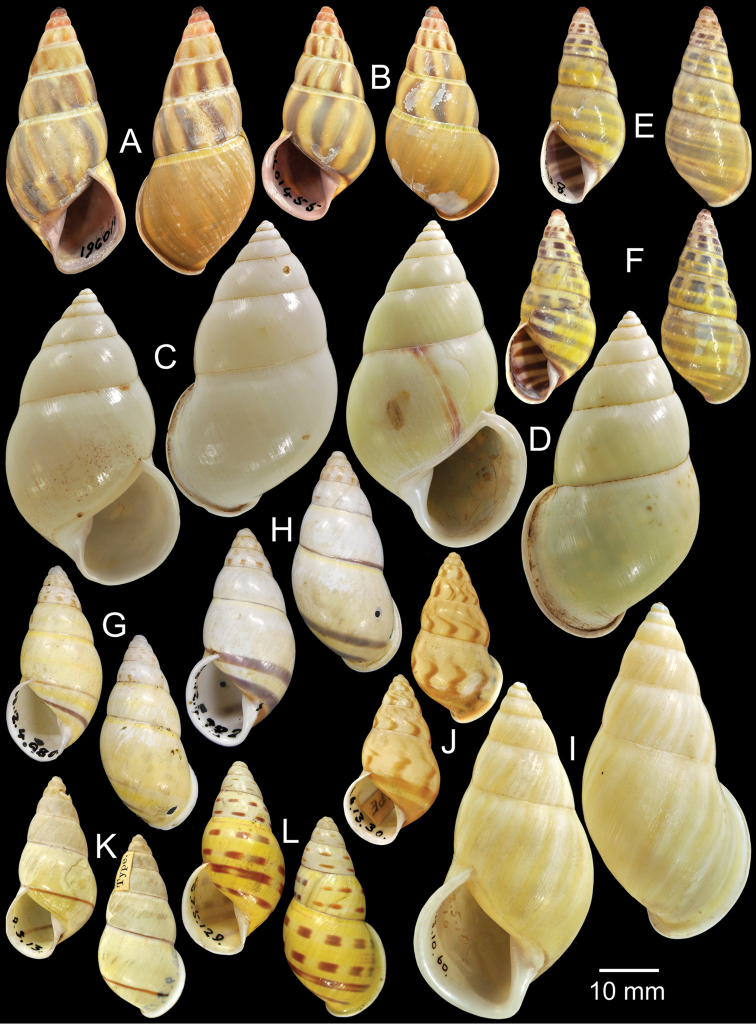
Type specimens of *Amphidromus* spp. **A–B** *Amphidromus glaucolarynx* **A** lectotype and **B** paralectotype **C–D** *Amphidromus perakensis globosus*, **C** lectotype and **D** paralectotype **E–F** *Amphidromus columellaris gloriosa* **E** lectotype and **F** paralectotype **G–H** *Amphidromus sinensis gracilis* **G** lectotype and **H** paralectotype **I** Possible syntype of *Amphidromus maculiferus garcilior* **J** Lectotype of *Amphidromus hamatus* **K** Holotype of *Amphidromus hosei* **L** Holotype of *Amphidromus inconstans*.

Lectotype NHMUK 19601454 (Fig. 8A; H=44.2 mm, W=20.1 mm), paralectotypes NHMUK 19601455 (1D + 2S, Fig. 8B).

#### Amphidromus sinensis globosa

Taxon classificationAnimaliaStylommatophoraCamaenidae

Nevill, 1878 [nomem nudum, ICZN (1999: Art. 12)].

Amphidromus sinensis var. globosa Nevill, 1878: 126 [*nomem nudum*].

##### Type locality.

Chittagong [now in Bangladesh].

##### Type material.

Original specimen NHMUK 1903.7.1.1921 (1S, H=25.1 mm, W=16.5 mm).

##### Remarks.

The name “*globosa*”, an unavailable name, was included in this catalog in order to indicate the history of the taxon. This name was introduced without a description or indication and therefore failed to conform to the ICZN guidelines (1999: Art. 12) and could not be made available by Nevill (1878). Later, this name was cited in Pilsbry (1900: 191) and Richardson (1985: 44). They cited this name without vaidating the taxon, and so this name could not be made available in subsequent works (ICZN 1999: Arts 11.5.2, 12).

We have surveyed for *Amphidromus* in western Thailand and collected a number of specimens with similar shell morphology to Nevill’s (1878) original specimen. It appear to be an undescribed species, therefore, we provide a species description and description of genitalia anatomy as Amphidromus (Syndromus) globonevilli Sutcharit and Panha, sp. n. (see description below).

#### Amphidromus perakensis globosus

Taxon classificationAnimaliaStylommatophoraCamaenidae

Fulton, 1903

Amphidromus perakensis var. globosus Fulton in Collinge, 1903: 211, 212.

##### Type locality.

Biserat, Jalor [Yala, Thailand].

##### Type material.

Lectotype (design. n.), NHMUK 1904.5.26.24 (Fig. 8C; H=50.0 mm, W=28.5 mm), paralectotypes NHMUK 1904.5.26.25-30 (4D adults + 2D juveniles, Fig. 8D).

##### Remarks.

This species was described based on specimens from Annandale and Robinson’s collection in the Malay Peninsula. Laidlaw and Solem (1961: 622) stated “… the location of the material is unknown.” We located seven specimens in the NHM general collections with an original label stating that they were purchased from Annandale and Robinson, with the locality “Biserat State of Jalor, Malay Peninsula”. We consider these specimens to be the syntypes. The specimen that most closely matches the original description is here designated as the lectotype.

Regarding the authorship of this name, Collinge (1903: 211, 212) clearly stated that H. Fulton provided him with the brief definition and the species name. Fulton, therefore is solely attributed the authorship (ICZN 1999: Art. 50.1.1).

#### Amphidromus columellaris gloriosa

Taxon classificationAnimaliaStylommatophoraCamaenidae

Fulton, 1896

Amphidromus columellaris var. gloriosa Bttg. Fulton 1896a: 79.

##### Type locality.

Sierah Island, Tenimber Laut [Tanimbar Islands, Indonesia].

##### Type material.

Lectotype (design. n.), NHMUK 1894.5.23.8 (Fig. 8E; H=32.5 mm, W=14.1 mm), paralectotypes NHMUK 1894.5.23.7 (Fig. 8F), SMF 7555 (3S).

##### Remarks.

Authorship was originally attributed to O. Boettger from a manuscript name. However, since O. Boettger did not write the description, the taxon is attributed to Fulton only. The brief original description clearly implied that it was based on more than one specimen. However, no illustration or measurements were provided, and the unique type was not designated in the original publication. Two specimens from NHM collection accompanied with Fulton’s handwritten label stating the taxon name and collection locality are considered to be syntypes. The specimen that most closely matches with the description is here designated as the lectotype to stabilise the name.

#### Amphidromus sinensis gracilis

Taxon classificationAnimaliaStylommatophoraCamaenidae

Fulton, 1896

Amphidromus sinensis var. gracilis Fulton, 1896a: 80, pl. 6, fig. 10.

##### Type locality.

Pegu, Burma [Bago, Myanmar].

##### Type material.

Lectotype NHMUK 1888.12.4.980 (Fig. 8G; H=26.7 mm, W=13.0 mm), paralectotypes NHMUK 1888.12.4.981–2 (2S, Fig. 8H).

#### Amphidromus maculiferus gracilior

Taxon classificationAnimaliaStylommatophoraCamaenidae

Fulton, 1896

Bulimus maculiferus var. β. Pfeiffer, 1853: 319. Küster and Pfeiffer 1854: pl. 40, fig. 9.
Amphidromus maculiferus var. gracilior Pfeiffer, Fulton 1896a: 74, 75.

##### Type locality.

Mindanao Island [Philippines].

##### Type material.

Possible syntype NHMUK 1842.5.10.60 (1S, Fig. 8I; H=56.3 mm, W=28.8 mm).

##### Remarks.

Fulton (1896a: 74, 75) attributed the authorship of this species to L. Pfeiffer. However, “Gracilior” in Pfeiffer (1853: 319) is only the first word of the description which is not a valid name (ICZN 1999: Art. 11.9). Therefore, Fulton (1896a: 74) is the sole author of this species.

The specimens that Pfeiffer used as the basis for “*Bulimus maculiferus* var. β.”, were examined and used by Fulton, and are acknowledged as the type series (ICZN 1999: Art 72.4). A single specimen in the NHM from H. Cuming’ s collection with Fulton’s handwritten labels bearing the taxon name and the locality “Mindanao, Philippines” is considered to be a possible syntype. This specimen corresponds closely with the figure in Küster and Pfeiffer (1854: pl. 40, fig. 9). However, the specimen is much smaller in size than the specimens quoted in Pfeiffer (1853: 319). Therefore, we treat the NHM specimen as a possible syntype.

#### Amphidromus hamatus

Taxon classificationAnimaliaStylommatophoraCamaenidae

Fulton, 1896

Amphidromus hamatus Fulton, 1896a: 84, pl. 5, fig. 13.

##### Type locality.

Labuan Island [Sabah, Malaysia].

##### Type material.

Lectotype NHMUK 1896.6.13.30 (Fig. 8J; H=27.7 mm, W=15.0 mm).

#### Amphidromus hosei

Taxon classificationAnimaliaStylommatophoraCamaenidae

Smith, 1895

Amphidromus hosei Smith, 1895: 115, pl. 3, fig. 20.

##### Type locality.

Meri, Sarawak.

##### Type material.

Holotype NHMUK 1894.9.3.13 (Figs 1F, 8K; H=30.6 mm, W=14.5 mm).

#### Amphidromus winteri inauris

Taxon classificationAnimaliaStylommatophoraCamaenidae

Fulton, 1896

Amphidromus winteri var. inauris (Bttg. MSS.) Fulton 1896a: 74, pl. 6, figs 12, 12a.

##### Type locality.

Java.

##### Type material.

**Figure 9.**
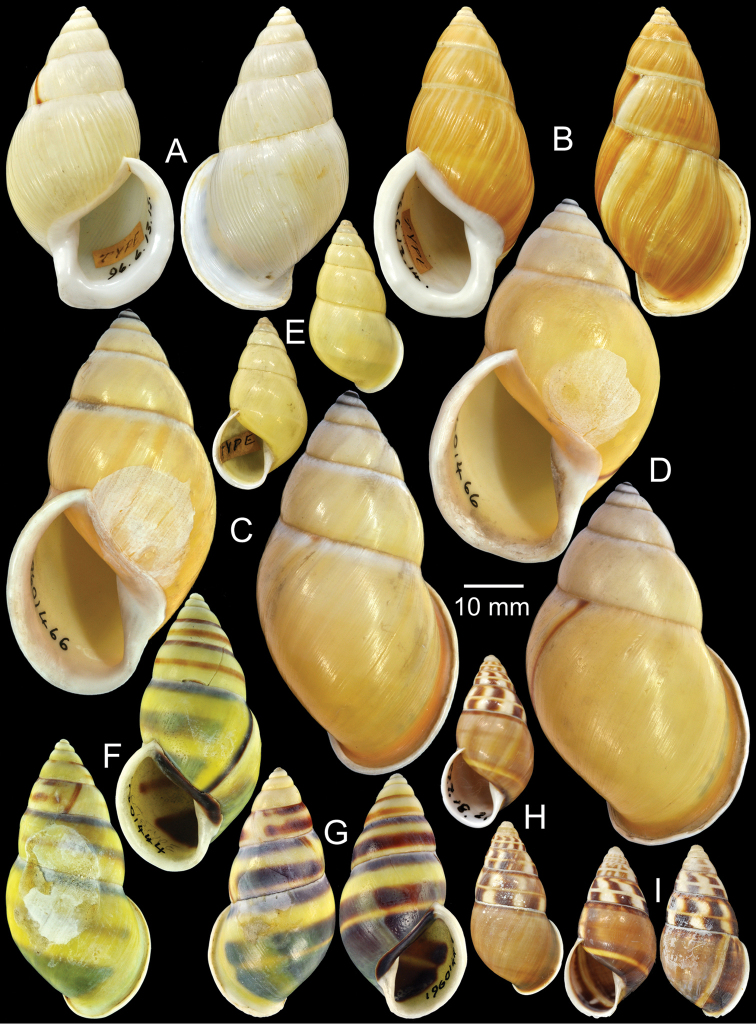
Type specimens of *Amphidromus* spp. **A–B** *Amphidromus winteri inauris* **A** lectotype and **B** paralectotype **C–D** *Amphidromus maculiferus inflata* **C** lectotype and **D** paralectotype **E** Holotype of *Amphidromus adamsii inornata* **F–G** *Amphidromus janus* **F** lectotype and **G** paralectotype **H–I** *Amphidromus filozonatus jucunda* **H** lectotype and **I** paralectotype.

Lectotype NHMUK 1896.6.13.13 (Fig. 9A; H=50.3 mm, W=27.5 mm), paralectotype NHMUK 1896.6.13.14 (1S, Fig. 9B), SMF 7638 (1S).

##### Remarks.

Fulton wrote “Bttg. MSS.” after the variety name, but did not appear to give O. Boettger credit for the description. Therefore, authorship is attributed to Fulton.

#### Amphidromus inconstans

Taxon classificationAnimaliaStylommatophoraCamaenidae

Fulton, 1898

Amphidromus inconstans Fulton, 1898: 10, text fig.

##### Type locality.

Alor (= Ombai) Island, Malayan Archipelago [East Nusa Tenggara, Indonesia].

##### Type material.

Holotype NHMUK 1898.7.5.129 (Fig. 8L; H=36.8 mm, W=18.8 mm), paratypes SMF 7563 (4S).

#### Amphidromus maculiferus inflata

Taxon classificationAnimaliaStylommatophoraCamaenidae

Fulton, 1896

Amphidromus maculiferus var. inflata Fulton, 1896a: 75.

##### Type locality.

Baranda Philippines Islands.

##### Type material.

Lectotype (design. n.), NHMUK 19601466/1 (Fig. 9C; H=64.2 mm, W=34.2 mm), paralectotypes NHMUK 19601466/2-3 (2S, Fig. 9D).

##### Remarks.

Three specimens originally from the Fulton collection with his handwritten label bearing the taxon and the type locality were located in the NHM collections. The largest shell (64.2 × 34.2 mm) clearly corresponds to the original description and Fulton’s measurements (66 × 38 mm) and so is here designated as the lectotype to stabilise the name.

#### Amphidromus adamsii inornata

Taxon classificationAnimaliaStylommatophoraCamaenidae

Fulton, 1896

Amphidromus adamsi var. inornata Fulton, 1896a: 83, pl. 5, fig. 6.

##### Type locality.

North Borneo.

##### Type material.

Lectotype NHMUK 1896.6.13.12 (Fig. 9E; H=27.3 mm, W=14.6 mm).

#### Amphidromus iunior

Taxon classificationAnimaliaStylommatophoraCamaenidae

Cilia, 2013

Amphidromus (Syndromus) iunior Cilia, 2013: 264–266, figs 1–6.

##### Type locality.

Mangili village, east part of Sumba Island, East Nusa Tenggara, Indonesia.

##### Type material.

Holotype MNHN 23265, paratypes FMNH 328120 (2S), MNHN 23266 (2S), NHMUK 20120044 (3S).

#### Amphidromus janus

Taxon classificationAnimaliaStylommatophoraCamaenidae

(Pfeiffer, 1854)

Bulimus janus Pfeiffer, 1854 [1852]: 85.

##### Type locality.

in Novis Hebridibus [New Hebrides].

##### Type material.

Lectotype NHMUK 19601444 (Fig. 9F; H=46.6 mm, W=24.0 mm), paralectotypes NHMUK 19601445 (1D + 1S, Fig. 9G).

##### Remarks.

The type locality “New Hebrides” seems to be an error, since this is beyond the known range of *Amphidromus*. Subsequent collections and reports confine the species distribution to Burma from the Tavoy and Mergui archipelagos (Nevill 1878, Pilsbry 1900, Gude 1914, Laidlaw and Solem 1961).

#### Amphidromus filozonatus jucunda

Taxon classificationAnimaliaStylommatophoraCamaenidae

Fulton, 1896

Amphidromus filozonatus var. jucunda Fulton, 1896a: 78, pl. 7, fig. 8.

##### Type locality.

Macassar, Celebes [Makassar, South Sulawesi, Indonesia].

##### Type material.

Lectotype NHMUK 1857.7.18.2/1 (Fig. 9H; H=28.0 mm, W=14.3 mm), paralectotype NHMUK 1857.7.18.2/2 (1S, Fig. 9I).

#### Amphidromus kalaoensis

Taxon classificationAnimaliaStylommatophoraCamaenidae

Fulton, 1896

Amphidromus kalaoensis Fulton, 1896b: 102.

##### Type locality.

Kalao Island [South Sulawesi, Indonesia].

##### Type material.

**Figure 10.**
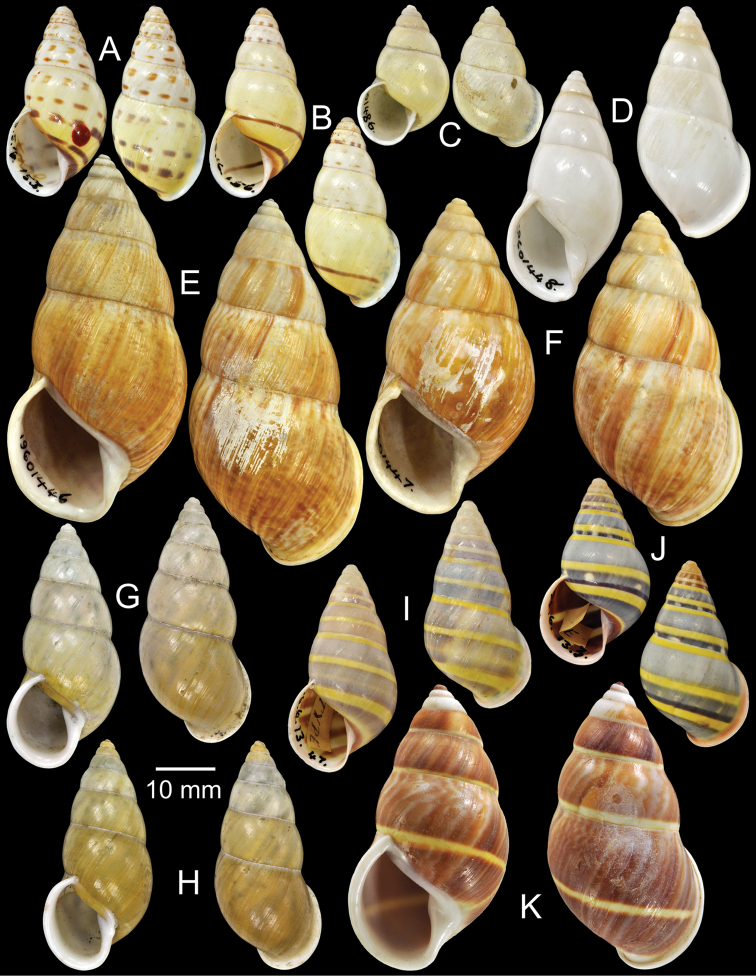
Type specimens of *Amphidromus* spp. **A–B** *Amphidromus kalaoensis* **A** holotype and **B** paratype **C** Lectotype of *Amphidromus lepidus* **D** Lectotype of *Amphidromus lindstedti* **E–F** *Amphidromus loricatus* **E** lectotype and **F** paralectotype **G–H** *Amphidromus sinistralis lutea* **G** lectotype and **H** paralectotype **I–J** *Amphidromus adamsii luteofasciatus* **I** lectotype and **J** paralectotype **K** Possible syntype of *Amphidromus melanomma*.

Holotype NHMUK 1896.5.16.153 (Fig. 10A; H=31.9 mm, W=15.6 mm), paratypes NHMUK 1896.5.16.154–6 (3S, Fig. 10B).

#### Amphidromus lepidus

Taxon classificationAnimaliaStylommatophoraCamaenidae

(Gould, 1856)

Bulimus lepidus Gould, 1856: 12.

##### Type locality.

Mergui Islands [Mergui Archipelago, Tanintharyi Region, Myanmar].

##### Type material.

Lectotype (design. n.), NHMUK 19601486 (Figs 2A, 10C; H=22.0 mm, W=14.0 mm).

##### Remarks.

Johnson (1964: 28, 29) indicated that some of the unlocated specimens from Gould’s type catalogue were probably in the NHM, since Gould presented some specimens to H. Cuming. No speceimens of *Bulimus lepidus* Gould, 1856 could be located by Johnson (1964: 100). There is a specimen in the NHM from the H. Cuming collection marked with “Type” and the locality “Mergui Islands” (Fig. 2A) which corresponds to the type locality, and the shell matches the measurements given in the original description (height 22.5 mm, width 12.5 mm). In addition, Fulton (1896a: 80) stated that “the type” of *Bulimus lepidus* is in the British Museum (now the NHM). This specimen is, therefore, considered as the syntype, and is here designated as the lectotype to stabilise the name. It is figured here for the first time since it was described.

#### Amphidromus lindstedti

Taxon classificationAnimaliaStylommatophoraCamaenidae

(Pfeiffer, 1857)

Bulimus lindstedti Pfeiffer, 1857c [1856]: 388.

##### Type locality.

Malacca.

##### Type material.

Lectotype NHMUK 19601448 (Fig. 10D; H=38.9 mm, W=18.4 mm).

##### Remarks.

Fulton (1896a: 85) described the type as being bleached in condition, and suggested that the specimen should have a color pattern if it were not bleached. We examined the lectotype but, in contrast, consider it to be entirely white in shell colour and not bleached. In addition, Laidlaw and Solem (1961) suggested that *Amphidromus quadrasi* Hidalgo, 1887 and *Amphidromus versicolor* Fulton, 1896 from the Philippines were probably junior synomyms of this species. With a unique straight columella, thickened parietal callus and elongated aperture, *Amphidromus lindstedti* (Pfeiffer, 1857) is clearly distinct from both speceis. However, new collections from precise localities will help elucidate whether this is a distinct species or a colour form of the other taxa.

#### Amphidromus loricatus

Taxon classificationAnimaliaStylommatophoraCamaenidae

(Pfeiffer, 1855)

Bulimus loricatus Pfeiffer, 1855 [1854]: 293.

##### Type locality.

unknown.

##### Type material.

Lectotype NHMUK 19601446 (Fig. 10E; H=61.5 mm, W=29.2 mm), paralectotype NHMUK 19601447 (1S, Fig. 10F).

##### Remarks.

The original description gave the type locality as “unknown”. However, the original label accompanying the lectotype states it was collected from Java. The type locality of this taxa is, therefore, confined to Java.

#### Amphidromus sinistralis lutea

Taxon classificationAnimaliaStylommatophoraCamaenidae

Fulton, 1896

Bulimus sinistralis var. B. Martens, 1867: 355, pl. 21, fig. 2b.
Amphidromus sinistralis var. lutea Martens, Fulton 1896a: 76.

##### Type locality.

Moluccas [probably in the area of Maluku and North Maluku, Indonesia].

##### Type material.

Lectotype (design. n.), NHMUK 20140752/1 (Fig. 10G; H=40.8 mm, W=18.9 mm), paralectotypes NHMUK 20140752/2–8 (7S, Fig. 10H).

##### Remarks.

Fulton (1896a) correctly nominated this name, but attributed the authorship to von Martens. However, von Martens (1867: 355) described the subspecific name as ‘B’ which is an invalid (ICZN 1999: Art. 11.9). Therefore, the authorship of this taxon should be attributed to Fulton.

The original description was very brief, without any measurements or illustrations, and did not indicate that a unique type was designated. The NHM holds a lot with eight shells from the Da Costa collection, with the original label stating “Fulton co-types” which are considered syntypes. The specimen that has a small label with Fulton’s handwritten glued inside the aperture is designated here as the lectotype to stabilise the name. The paralectotypes are the other seven specimens from the same lot.

#### Amphidromus adamsii luteofasciata

Taxon classificationAnimaliaStylommatophoraCamaenidae

Fulton, 1896

Amphidromus adamsi var. luteofasciata Fulton, 1896a: 82, pl. 5, figs 2, 2a.

##### Type locality.

Banguey Island [Sabah, Malaysia].

##### Type material.

Lectotype NHMUK 1896.6.13.47 (Figs 2B, 10I; H=34.4 mm, W=17.2 mm), paralectotypes NHMUK 1896.6.13.3 (1S, Figs 2B, 10J), SMF 7549 (2S).

#### Amphidromus contrarius maculata

Taxon classificationAnimaliaStylommatophoraCamaenidae

Fulton, 1896

Amphidromus contrarius var. maculata Fulton, 1896a: 78, pl. 7, fig. 4.

##### Type locality.

Macassar [Makassar, South Sulawesi, Indonesia].

##### Type material.

**Figure 11.**
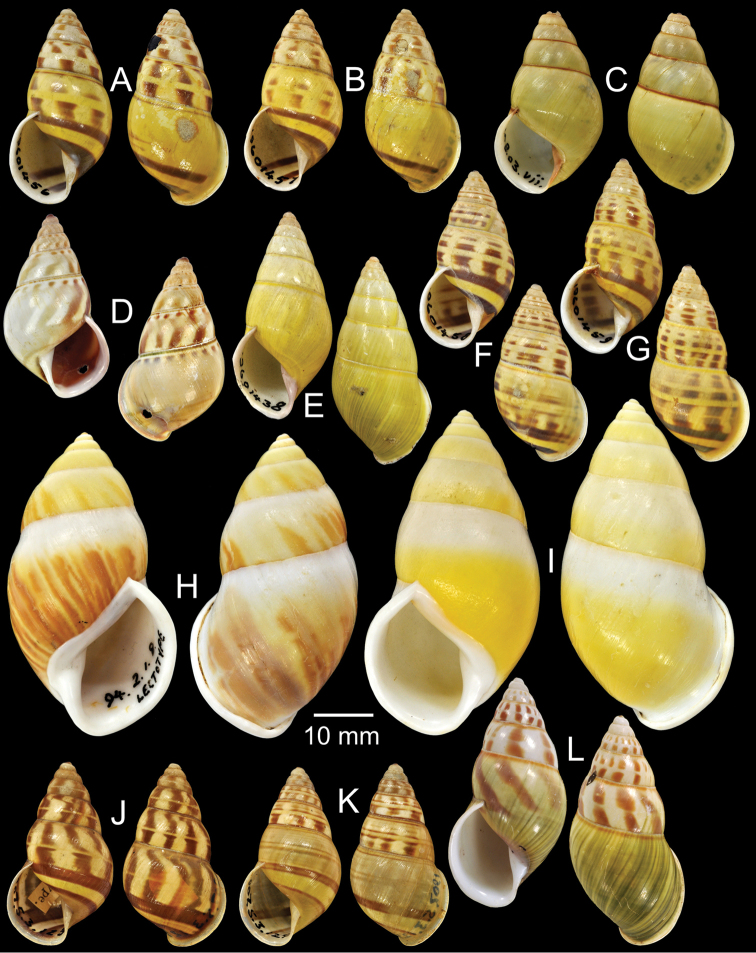
Type specimens of *Amphidromus* spp. **A–B** *Amphidromus contrarius maculata* **A** lectotype and **B** paralectotype **C** Lectotype of *Amphidromus masoni* **D** Lectotype of *Amphidromus moniliferus* **E** Lectotype of *Amphidromus mouhoti* **F–G** *Amphidromus contrarius multifasciata* **F** lectotype and **G** paralectotype **H–I** *Amphidromus perversus natunensis* **H** lectotype and **I** paralectotype **J–K** *Amphidromus niasensis* **J** lectotype and **K** paralectotype **L** Holotype of *Amphidromus nicobarica*.

Lectotype NHMUK 19601456 (Fig. 11A; H=32.1 mm, W=16.9 mm), paralectotype NHMUK 19601457 (1S, Fig. 11B), SMF 28294 (1S).

#### Amphidromus masoni

Taxon classificationAnimaliaStylommatophoraCamaenidae

(Godwin-Austen, 1876)

Bulimus masoni Godwin-Austen, 1876: 316.

##### Type locality.

Dihiri Parbat, 2000 feet [Dafla Hills, Assam, India].

##### Type material.

Lectotype (design. n.), NHMUK 1903.7.1.1908 (Figs 2C, 11C; H=30.7 mm, W=17.7 mm).

##### Remarks.

Godwin-Austen (1876) stated that there were two specimens in the type series. Only a single specimen from the Godwin-Austen type lot remains in the NHM collections (Fig. 2C). Laidlaw and Solem (1961: 639) considered this specimen to be the holotype. This should be interpreted as an inadvertant lectotype designation (ICZN 1999: Art. 74.6). The apex of the lectotype has been damaged at around the second and third whorls and the shell height is much smaller than it would have been if undamaged.

#### Amphidromus melanomma

Taxon classificationAnimaliaStylommatophoraCamaenidae

(Pfeiffer, 1852)

Bulimus melanomma Pfeiffer, 1852: 95.

##### Type locality.

insulis Moluccis [= the islands of the Moluccas].

##### Type material.

Possible syntype NHMUK 20140753/1 (Figs 2D, 10K; H=47.8 mm, W=26.3 mm).

##### Remarks.

The original description by Pfeiffer (1852: 95) did not give an illustration of the species but a set of measurements were provided. Küster and Pfeiffer (1854: 135, 136, pl. 39, figs 28, 29; pl. 41, figs 1, 2, 7, 8) re-published the description, and figured the nominal species and included two varietal forms.

The NHM holds a lot that has an original label in Pfeiffer’s handwriting giving the species name (in blue ink) and the collection locality of “Malacca”. The words “*Bulimus melanoma* var γ Pfr. Mon. Hel. III p. 310”, not written by Pfeiffer, were added to the label at a later time (Fig. 2D). The specimen illustrated in Küster and Pfeiffer (1854: pl. 39, figs 27, 28) is recognized by the sinistral shell, with a yellow peripheral band on the periphery of the last whorl and the dimensions are very close to those given in the original description. Since the lot contains two other dextral specimens which were not mentioned in the original description, we refrain from designating this as a lectotype, considering the sinistral specimen to be a possible syntype. The other two dextral shells (NHMUK 20140753/2-3) that are contained in the lot are excluded from the type series (ICZN 1999: Art. 72.4.1).

#### Amphidromus moniliferus

Taxon classificationAnimaliaStylommatophoraCamaenidae

(Gould, 1846)

Bulimus moniliferus Gould, 1846: 99.

##### Type locality.

Tavoy [Dawei, Tanintharyi Region, Myanmar].

##### Type material.

Lectotype (design. n.), NHMUK 20120009 (Figs 2E, 11D; H=29.3 mm, W=16.5 mm).

##### Remarks.

Gould noted that he received several specimens (dextral and sinistral) from F. Mason, and he wrote his original description from the sinistral specimen (Gould 1846). However, in the catalogue of Gould’s type specimens, Johnson (1964) could not locate any type material of *Bulimus moniliferus*. A single specimen was found in the NHM from the H. Cuming collection with “Type” written on it and the locality “Tavoy” which corresponds to the type locality in the original description (Fig. 2E). In addition, F. Mason, the original collector who presented specimens to Gould, mentioned that local people (Karen ladies) often strung the shells of *Amphidromus atricallosus* and others congeners from their necklaces (Mason 1850: 400). Evidence of a hole remains on the basal lip of the lectotype of *Bulimus atricallosus* (MCZ 169050) and in the NHM type specimen of *Bulimus moniliferus* Gould, 1846. This specimen is, therefore, designated as the lectotype to stabilise the name.

#### Amphidromus mouhoti

Taxon classificationAnimaliaStylommatophoraCamaenidae

(Pfeiffer, 1861)

Bulimus mouhoti Pfeiffer, 1861: 194.

##### Type locality.

Siam [Thailand].

##### Type material.

Lectotype NHMUK 19601438 (Fig. 11E; H=34.2 mm, W=16.1 mm).

#### Amphidromus contrarius multifasciata

Taxon classificationAnimaliaStylommatophoraCamaenidae

Fulton, 1896

Amphidromus contrarius var. multifasciata Fulton, 1896a: 78, pl. 7, fig. 5.

##### Type locality.

Cambodia.

##### Type material.

Lectotype NHMUK 19601458 (Fig. 11F; H=29.4 mm, W=15.1 mm); paralectotypes NHMUK 19601459 (2S, Fig. 11G).

#### Amphidromus perversus natunensis

Taxon classificationAnimaliaStylommatophoraCamaenidae

Fulton, 1896

Amphidromus perversus var. natunensis Fulton, 1896a: 69.

##### Type locality.

Natuna Islands [Indonesia].

##### Type material.

Lectotype NHMUK 1894.2.1.8 (Fig. 11H; H=51.7 mm, W=26.9 mm), paralectotypes NHMUK 1894.2.1.9–19 (5D + 6S, Fig. 11I).

#### Amphidromus niasensis

Taxon classificationAnimaliaStylommatophoraCamaenidae

Fulton, 1907

Amphidromus niasensis Fulton, 1907: 151–152, pl. 9, fig. 9.

##### Type locality.

Nias Island, Sumatra.

##### Type material.

Lectotype NHMUK 1907.5.3.123 (Fig. 11J; H=30.1 mm, W=16.5 mm), paralectotype NHMUK 1907.5.3.124 (1S, Fig. 11K).

#### Amphidromus andamanicus nicobarica

Taxon classificationAnimaliaStylommatophoraCamaenidae

Godwin-Austen, 1895

Amphidromus andamanicus var. nicobarica Godwin-Austen, 1895: 443, 450.

##### Type locality.

Katchall [island in Andaman and Nicobar Islands, India].

##### Type material.

Holotype NHMUK 1888.8.6.31 (Figs 2F, 11L; H=38.4 mm, W=19.0 mm).

##### Remarks.

Godwin-Austen clearly stated that this taxon was described based on only one specimen. Therefore a single specimen ex. Röepstorff (Fig. 2F) in the NHM collections is recognized as the holotype fixed by monotypy (ICZN 1999: Art. 73.1.2).

#### Amphidromus maculiferus obscura

Taxon classificationAnimaliaStylommatophoraCamaenidae

Fulton, 1896

Amphidromus maculiferus var. obscura Fulton, 1896a: 75.

##### Type locality.

Mindanao Island.

##### Type material.

**Figure 12.**
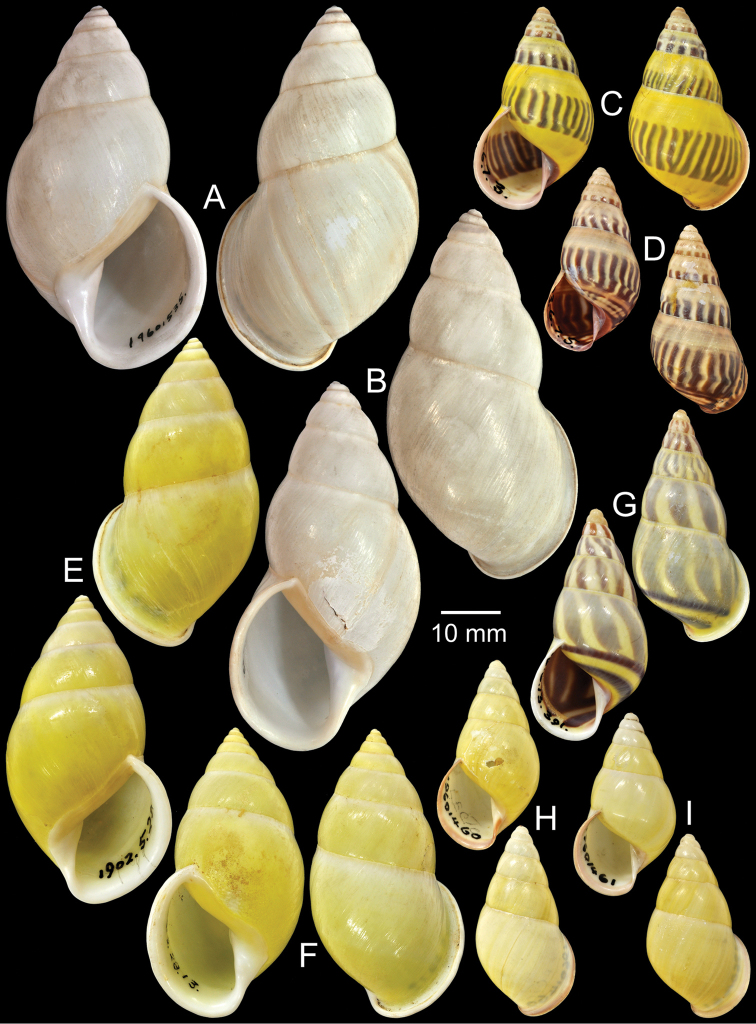
Type specimens of *Amphidromus* spp. **A–B** *Amphidromus maculiferus obscura* **A** lectotype and **B** paralectotype **C–D** *Amphidromus adamsii ornata* **C** lectotype and **D** paralectotype **E–F** *Amphidromus perakensis*, **E** lectotype and **F** paralectotype **G** Holotype of *Amphidromus pictus* **H–I** *Amphidromus placidus* **H** lectotype and **I** paralectotype.

Lectotype (design. n.), NHMUK 19601535/1 (Fig. 12A; H=61.4 mm, W=31.5 mm), paralectotypes NHMUK 19601535/2-3 (1D + 1S, Fig. 12B).

##### Remarks.

The original description was based on more than one specimen, since Fulton stated “…remarkable that this is the only form of *maculiferus* of which *dextral* specimens have been found…”. A unique type was not indicated in the original description. The NHM holds a lot that consists of three specimens (2D, 1S) from the H. Cuming collection with an original label in Fulton’s handwriting. The dextral specimen closely matches with the original description and is here designated as the lectotype to stabilise the name.

#### Amphidromus adamsii ornata

Taxon classificationAnimaliaStylommatophoraCamaenidae

Fulton, 1896

Amphidromus adamsi var. ornata Fulton, 1896a: 82, 83, pl. 5, fig. 14.

##### Type locality.

Banguey Island, Borneo [Sabah, Malaysia].

##### Type material.

Lectotype NHMUK 1893.6.7.3 (Fig. 12C; H=33.8 mm, W=19.4 mm), paralectotypes NHMUK 1893.6.7.4–5 (2S, Fig. 12D).

#### Amphidromus perakensis

Taxon classificationAnimaliaStylommatophoraCamaenidae

Fulton, 1901

Amphidromus perakensis Fulton, 1901: 104, pl. 9, figs 8–10.

##### Type locality.

Perak [Peninsular Malaysia].

##### Type material.

Lectotype NHMUK 1902.5.28.12 (Fig. 12E; H=51.0 mm, W=26.9 mm), paralectotypes NHMUK 1902.5.28.13 (1S, Fig. 12F), SMF 7595 (3D + 2S).

#### Amphidromus pictus

Taxon classificationAnimaliaStylommatophoraCamaenidae

Fulton, 1896

Amphidromus pictus Fulton, 1896a: 85, pl. 5, fig. 8.

##### Type locality.

Kina Balu, North Borneo.

##### Type material.

Lectotype NHMUK 96.6.13.391 (Fig. 12G; H=38.1 mm, W=18.6 mm).

#### Amphidromus placidus

Taxon classificationAnimaliaStylommatophoraCamaenidae

Fulton, 1896

Amphidromus placidus Fulton, 1896a: 84, pl. 5, fig. 11.

##### Type locality.

East Boneo.

##### Type material.

Lectotype NHMUK 19601460 (Fig. 12H; H=31.5 mm, W=16.2 mm), paralectotypes NHMUK 19601461 (2S, Fig. 12I).

#### Amphidromus poecilochroa

Taxon classificationAnimaliaStylommatophoraCamaenidae

Fulton, 1896

Amphidromus poecilochroa Fulton, 1896a: 77, pl. 6, fig. 7.

##### Type locality.

Sumbawa Island [West Nusa Tenggara, Indonesia].

##### Type material.

**Figure 13.**
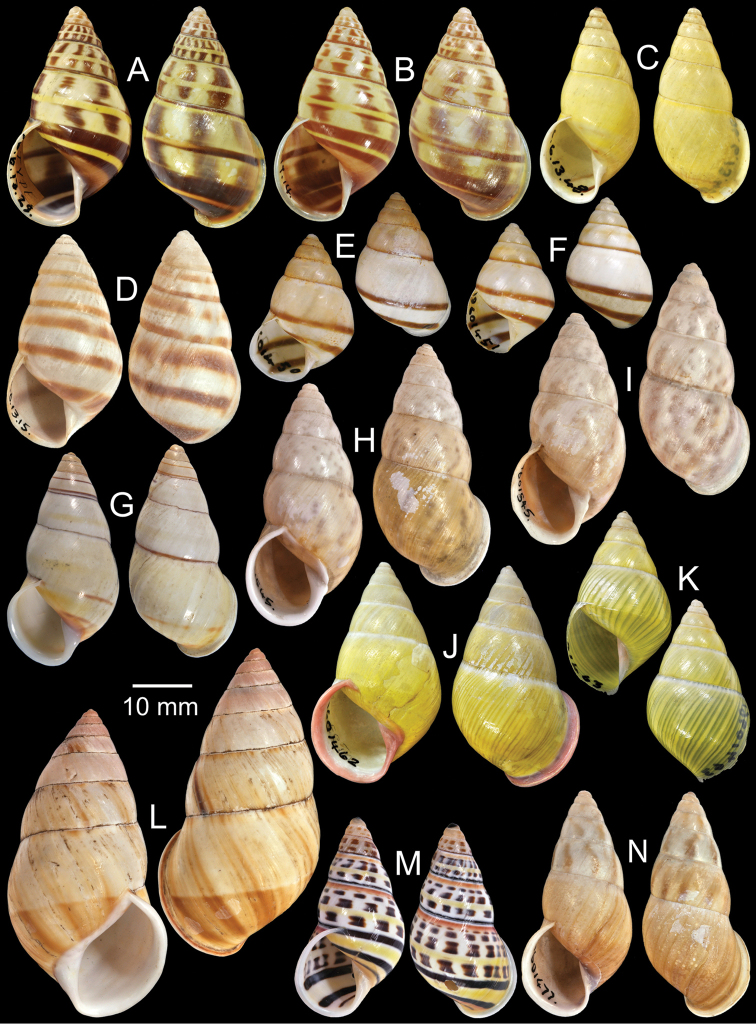
Type specimens of *Amphidromus* spp. **A–B** *Amphidromus poecilochroa* **A** lectotype and **B** paralectotype **C** Holotype of *Amphidromus flavus proxima* **D** Syntype of *Amphidromus robustus* **E–F** *Amphidromus roemeri* **E** lectotype and **F** paralectotype **G** Paralectotype of *Amphidromus laevus romaensis* **H–I** *Amphidromus sinistralis rosea* **H** lectotype and **I** paralectotype **J–K** *Amphidromus roseolabiatus* **J** lectotype and **K** paralectotype **L** Paralectotype of *Amphidromus annamiticus roseotincta* **M** Paratype of *Amphidromus rottiensis* **N** Probable syntype of *Amphidromus adamsii rubiginosa*.

Lectotype NHMUK 1896.6.13.29 (Fig. 13A; H=36.1 mm, W=20.0 mm), paralectotypes NHMUK 1895.12.19.13-14 (2S, Fig. 13B), SMF 7594 (2S).

#### Amphidromus flavus proxima

Taxon classificationAnimaliaStylommatophoraCamaenidae

Fulton, 1896

Amphidromus flavus var. proxima , Fulton 1896a: 81, pl. 6, fig. 4.

##### Type locality.

unknown.

##### Type material.

Holotype NHMUK 1896.6.13.48 (Fig. 13C; H=32.6 mm, W=16.1 mm).

#### Amphidromus robustus

Taxon classificationAnimaliaStylommatophoraCamaenidae

Fulton, 1896

Amphidromus robustus Fulton, 1896a: 73.

##### Type locality.

Java.

##### Type material.

Syntype NHMUK 1896.6.13.15 (1D juvenile, Fig. 13D; H=35.3 mm, W=18.8 mm).

##### Remarks.

The specimen from Fulton’s collection with an accompanied label bearing a handwritten taxon and locality is considered to be a syntype (ICZN 1999: Art. 72.4). However, in the original description, Fulton provided the measurements of an adult specimen, yet only a juvenile specimen was located in the NHM.

#### Amphidromus roemeri

Taxon classificationAnimaliaStylommatophoraCamaenidae

(Pfeiffer, 1863)

Bulimus römeri Pfeiffer, 1863 [1862]: 274, pl. 36, fig. 4.

##### Type locality.

Lao Mountains, Cambodja [=Cambodia].

##### Type material.

Lectotype NHMUK 19601450 (Fig. 13E; H=23.1 mm, W=15.0 mm), paralectotypes NHMUK 19601451 (2S juveniles, Fig. 13F).

#### Amphidromus laevus romaensis

Taxon classificationAnimaliaStylommatophoraCamaenidae

Rolle, 1903

Amphidromus laevus var. romaensis Rolle, 1903: 157.

##### Type locality.

Insel Roma, Timor.

##### Type material.

Lectotype (designated by Zilch 1953: 133, pl. 22, fig. 11), SMF 7574a, paralectotypes SMF 7574b-c, NHMUK 1908.7.6.78 (1S, Fig. 13G).

##### Remarks.

The original description was very brief and H. Rolle never designated a unique name-bearing type. Later, Zilch (1953) designated the lectotype from H. Rolle’s collection in the Senckenberg Museum. The NHM registration records show that a specimen was purchased from Sowerby and Fulton’s collection with the original label stating “Co-type” and giving the locality “Roma I.”. Therefore, we consider this specimen to be a paralectotype.

#### Amphidromus sinistralis rosea

Taxon classificationAnimaliaStylommatophoraCamaenidae

Fulton, 1896

Amphidromus sinistralis var. rosea Fulton, 1896a: 76.

##### Type locality.

Nördliches Celebes [Northern Sulawesi, Indonesia].

##### Type material.

Lectotype (design. n.), NHMUK 19601545/1 (Fig. 13H; H=40.1 mm, W=19.2 mm), paralectotypes NHMUK 19601545/2-3 (2S, Fig. 13I).

##### Remarks.

Fulton attributed the authorship to von Martens. However, von Martens (1867: 356, pl. 21, fig. 2c) only describe this varietal form with a letter “*Bulimus sinistralis* var. C.” which is not a valid name (ICZN 1999: Art. 11.9). Therefore, Fulton (1896a: 76) is the sole author of this species.

The NHM holds a lot with Fulton’s handwritten labels bearing the taxon and type locality. The specimen that corresponds most closely with the original description is designated as the lectotype.

#### Amphidromus roseolabiatus

Taxon classificationAnimaliaStylommatophoraCamaenidae

Fulton, 1896

Amphidromus roseolabiatus Fulton, 1896a: 89, pl. 6, fig. 8.

##### Type locality.

Siam [Thailand].

##### Type material.

Lectotype NHMUK 19601462 (Fig. 13J; H=36.5 mm, W=20.7 mm), paralectotype NHMUK 19601463 (1S, Fig. 13K).

#### Amphidromus annamiticus roseotincta

Taxon classificationAnimaliaStylommatophoraCamaenidae

Möllendorff, 1894

Amphidromus annamiticus var. roseotincta Möllendorff, 1894: 150.

##### Type locality.

near Chaya [Chaiya, Suratthani, Thailand].

##### Type material.

Lectotype (designated by Zilch 1953: 135, pl. 23, fig. 26), SMF 7546, paralectotypes SMF 7547 (7D), SMF 28241 (10D), SMF 82356 (2D), SMF 82357 (4S), NHMUK 1894.2.26.45–46 (2D, Fig. 13L).

##### Remarks.

Möllendorff (1894) provided a very brief definition of the taxon without figures. The type locality as written on the lectotype label was “Tschaya”. The NMH possess a lot of two shells purchased from H. Rolle, which are considered to be probable paralectotypes.

#### Amphidromus rottiensis

Taxon classificationAnimaliaStylommatophoraCamaenidae

Chan & Tan, 2010

Amphidromus rottiensis Chan, Tan & Abbas, 2008: 2, 3, fig. 1. [*nomen nudum*, ICZN (1999: Arts 8.6 and 11.1)].
Amphidromus rottiensis Chan & Tan, 2010: 246, fig. 1G–I.

##### Type locality.

Southwest central plateau portion (Busalangga) of Rotti Island (Pulau Rote), Indonesia.

##### Type material.

Holotype MZBGst.15.047 (Ex NHMUK 20080621), paratypes NHMUK 20080622 (3S, Fig. 13M).

##### Remarks.

Chan et al. (2008) described “*rottiensis*” in the Occasional Molluscan Papers which does not fulfil the ICZN (1999: Art. 8.6) guidelines, and could not be made available (ICZN 1999: Art. 11.1). However, the same species name was later published correctly (ICZN 1999: Art. 8) and made available in Chan and Tan (2010).

#### Amphidromus adamsii rubiginosa

Taxon classificationAnimaliaStylommatophoraCamaenidae

Fulton, 1896

Amphidromus adamsi var. rubiginosa Fulton, 1896a: 84.

##### Type locality.

N. Borneo.

##### Type material.

2 probable syntypes NHMUK 19601477 (2S, Fig. 13N).

##### Remarks.

There are two specimens from H. Cuming’s collection accompanied with Fulton’s handwritten label stating the taxon name. The type locality in the original publication was given as N. Borneo, but this lot has no locality. However, these specimens closely match the original description, especially in colour pattern and so it seems likely that these were indeed the shells that Fulton based the species description upon. Therefore, on this basis, we consider these specimens to be probable syntypes.

#### Amphidromus adamsii rufocincta

Taxon classificationAnimaliaStylommatophoraCamaenidae

Fulton, 1896

Amphidromus adamsi var. rufocincta Fulton, 1896a: 83, pl. 5, fig. 1.

##### Type locality.

Borneo.

##### Type material.

**Figure 14.**
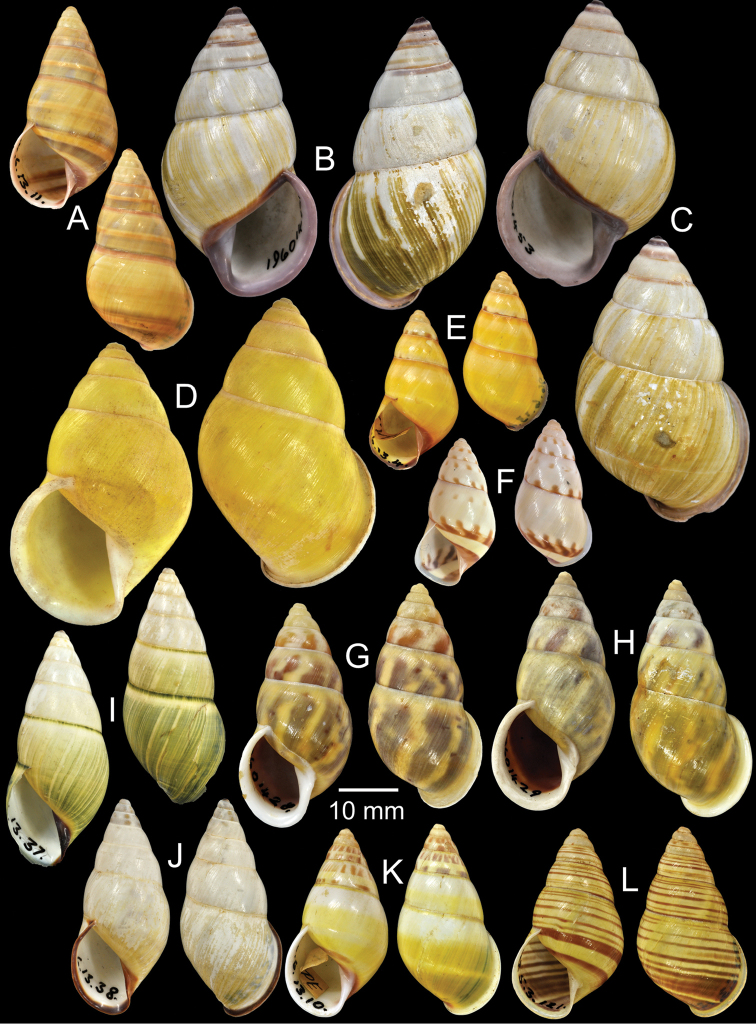
Type specimens of *Amphidromus* spp. **A** Lectotype of *Amphidromus adamsii rufocincta* **B–C** *Amphidromus schomburgki* **B** lectotype and **C** paralectotype **D** Paratype of *Amphidromus webbi simalurensis* **E** Lectotype of *Amphidromus adamsii simplex* **F** Paralectotype of *Amphidromus singalangensis* **G–H** *Amphidromus sinistralis* **G** lectotype and **H** paralectotype **I–J** *Amphidromus smithii* **I** lectotype and **J** paralectotype **K** Lectotype of *Amphidromus quadrasi solida* **L** Lectotype of *Amphidromus sowerbyi*.

Lectotype NHMUK 1896.6.13.11 (Fig. 14A; H=34.2 mm, W=17.7 mm).

#### Amphidromus schomburgki

Taxon classificationAnimaliaStylommatophoraCamaenidae

(Pfeiffer, 1860)

Bulimus schomburgki Pfeiffer, 1860: 137, pl. 51, fig. 9.

##### Type locality.

Siam [Thailand].

##### Type material.

Lectotype NHMUK 19601452 (Fig. 14B; H=48.0 mm, W=25.5 mm), paralectotypes NHMUK 19601453 (1D + 1S, Fig. 14C).

#### Amphidromus webbi simalurensis

Taxon classificationAnimaliaStylommatophoraCamaenidae

Laidlaw, 1954

Amphidromus webbi var. simalurensis Laidlaw, 1954: 78, 79.

##### Type locality.

Soea Lamatau, Simalur Island [Simeulue Island, Aceh, Indonesia].

##### Type material.

Holotype in RMNH, paratype NHMUK 1957.11.18.2 (1S, Fig. 14D).

#### Amphidromus adamsii simplex

Taxon classificationAnimaliaStylommatophoraCamaenidae

Fulton, 1896

Amphidromus adamsi var. simplex Fulton, 1896a: 83, pl. 5, fig. 12.

##### Type locality.

Banguey Island [Sabah, Malaysia].

##### Type material.

Lectotype NHMUK 1896.6.13.4 (Fig. 14E; H=26.1 mm, W=13.4 mm).

#### Amphidromus singalangensis

Taxon classificationAnimaliaStylommatophoraCamaenidae

Rolle, 1908

Amphidromus singalangensis Rolle, 1908: 67.

##### Type locality.

Ostabhang des Singalang, West Sumatra [Eastern slope of Mount Singgalang, West Sumatra, Indonesia].

##### Type material.

Lectotype (designated by Zilch 1953: 133, pl. 23, fig. 20), SMF 7671, paralectotypes NHMUK 1908.7.6.85-86 (2S, Fig. 14F), SMF 7672 (5S).

##### Remarks.

The lectotype was designated from H. Rolle’s collection (Zilch 1953: 133, pl. 23, fig. 20). The NHM holds one lot of 2 specimens from the type series, labeled as “co-type”, and these are considered paralectotypes.

#### Amphidromus sinistralis

Taxon classificationAnimaliaStylommatophoraCamaenidae

(Reeve, 1849)

Bulimus sinistralis Reeve, 1849: *Bulimus*, plate 81 species 603, fig. 603.

##### Type locality.

Java.

##### Type material.

Lectotype NHMUK 19601428 (Fig. 14G; H=37.3 mm, W=18.5 mm), paralectotypes NHMUK 19601429 (2S, Fig. 14H).

#### Amphidromus smithii

Taxon classificationAnimaliaStylommatophoraCamaenidae

Fulton, 1896

Amphidromus smithii Fulton, 1896a: 88, 89, pl. 7, figs 12, 12a.

##### Type locality.

Annam [Central Vietnam].

##### Type material.

Lectotype NHMUK 1896.6.13.37 (Fig. 14I; H=39.2 mm, W=16.5 mm), paralectotype NHMUK 1896.6.13.38 (1S, Fig. 14J).

#### Amphidromus quadrasi solida

Taxon classificationAnimaliaStylommatophoraCamaenidae

Fulton, 1896

Amphidromus quadrasi var. solida Fulton, 1896a: 86, pl. 5, fig. 16.

##### Type locality.

Palawan [Philippines].

##### Type material.

Lectotype NHMUK 1896.6.13.10 (Fig. 14K; H=31.5 mm, W=16.3 mm).

#### Amphidromus sowerbyi

Taxon classificationAnimaliaStylommatophoraCamaenidae

Fulton, 1907

Amphidromus sowerbyi Fulton, 1907: 152, pl. 9, fig. 10.

##### Type locality.

Nias Island, Sumatra.

##### Type material.

Lectotype NHMUK 1907.5.3.121 (Fig. 14L; H=31.1 mm, W=17.2 mm).

#### Amphidromus adamsii subunicolor

Taxon classificationAnimaliaStylommatophoraCamaenidae

Fulton, 1896

Amphidromus adamsi var. subunicolor Fulton, 1896a: 82, pl. 5, fig. 5.

##### Type locality.

Banguey Island [Sabah, Malaysia].

##### Type material.

**Figure 15.**
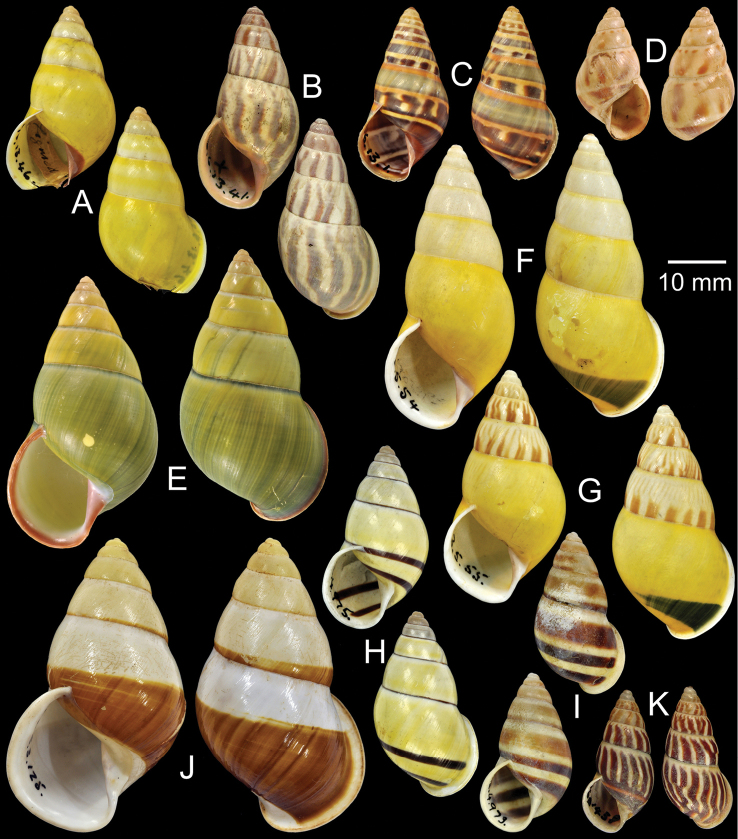
Type specimens of *Amphidromus* spp. **A** Lectotype of *Amphidromus adamsii subunicolor* **B** Lectotype of *Amphidromus sumbaensis* **C** Lectotype of *Amphidromus adamsii superba* **D** Possible syntype of *Amphidromus theobaldianus* **E** Paralectotype of *Amphidromus smithi ventrosulus* **F–G** *Amphidromus versicolor* **F** lectotype and **G** paralectotype **H–I** *Amphidromus sinensis vicaria* **H** lectotype and **I** paralectotype **J** Holotype of *Amphidromus webbi* **K** Lectotype of *Amphidromus zebrinus*.

Lectotype NHMUK 1896.6.13.46 (Fig. 15A; H=30.1 mm, W=16.8 mm).

##### Remarks.

Fulton attributed the authorship of this variety to von Martens (1867: 357). However, von Martens only describe this varietal form with a letter “*Bulimus adamsii* var. D.”, which is not a valid name (ICZN 1999: Art. 11.9). Later “*subunicolor*” was appropriately described and figured in Fulton (1896a). The basal lip or bottom of the aperture of the lectotype was damaged and so the shell height given here is much smaller than the actual specimen size.

#### Amphidromus sumbaensis

Taxon classificationAnimaliaStylommatophoraCamaenidae

Fulton, 1896

Amphidromus sumbaensis Fulton, 1896a: 102.

##### Type locality.

Sumba (Soemba) Island [Sumba Island, East Nusa Tenggara, Indonesia].

##### Type material.

Lectotype NHMUK 96.6.13.41 (Fig. 15B; H=34.1 mm, W=16.6 mm), paralectotype NHMUK 1896.6.13.42 (1S).

#### Amphidromus adamsii superba

Taxon classificationAnimaliaStylommatophoraCamaenidae

Fulton, 1896

Amphidromus adamsi var. superba Fulton, 1896a: 83, pl. 5, fig. 10.

##### Type locality.

Banguey Island [Sabah, Malaysia].

##### Type material.

Lectotype NHMUK 1896.6.13.1 (Fig. 15C; H=29.4 mm, W=14.4 mm).

#### Amphidromus theobaldianus

Taxon classificationAnimaliaStylommatophoraCamaenidae

(Benson, 1857)

Bulimus theobaldianus Benson, 1857: 329, 330.

##### Type locality.

Yanglaw, Tenasserim [in the area of Tanintharyi Region, Myanmar].

##### Type material.

Possible syntype NHMUK 1907.11.21.64 (1D juvenile; Fig. 15D; H=22.3 mm, W=12.4 mm).

##### Remarks.

The original description seems to be based on one specimen and a single set of measurements was given. Benson (1857: 329) stated “peristomate tenui?” [=peristome thin?], and Theobald (1876: 187) also stated “…described by Benson from an imperfect example”, which we have interpreted as meaning that the type specimen is an immature shell. The NHM holds a lot containing a juvenile specimen figured in Hanley and Theobald (1870: pl. 19, fig. 10), and the label states “from Hanley coll. figd in Con. Ind. pl. 19, fig. 10”. The collection locality states “Tenasserim” which agrees with the original description. However, this specimen is larger than the dimensions given, so we refrain from designating it as the lectotype.

#### Amphidromus smithi ventrosulus

Taxon classificationAnimaliaStylommatophoraCamaenidae

Möllendorff, 1900

Amphidromus smithi ventrosulus Möllendorff, 1900: 132, 133.

##### Type locality.

Phuc-son, Annam [Tan Yen District, Bac Giang Province, northeastern Vietnam].

##### Type material.

Lectotype (designated by Zilch 1953: 133, pl. 23, fig. 19), SMF 7643 (1S), paralectotypes SMF 7642/6 (6S), NHMUK 1902.3.22.20-21 (2S, Fig. 15E).

##### Remarks.

Möllendorff indicated that the specimens examined in the original description were from H. Fruhstorfer’s collection. The lectotype was designated by Zilch (1953: 133) and is housed in the Senckenberg Museum. The NHM registration records show that the two specimens were purchased from H. Fruhstorfer. The specimen locality is “Annam” which matches with the type locality. We therefore consider these specimens to be paralectotypes.

#### Amphidromus versicolor

Taxon classificationAnimaliaStylommatophoraCamaenidae

Fulton, 1896

Amphidromus versicolor Fulton, 1896a: 86.

##### Type locality.

Balabac [Balabac Island, Palawan, Philippines].

##### Type material.

Lectotype NHMUK 1893.3.5.54 (Fig. 15F; H=48.7 mm, W=21.8 mm), paralectotype NHMUK 1893.3.5.55 (1S, Fig. 15G).

#### Amphidromus sinensis vicaria

Taxon classificationAnimaliaStylommatophoraCamaenidae

Fulton, 1896

Amphidromus sinensis var. vicaria Fulton, 1896a: 80.

##### Type locality.

Pegu [Bago, northeast of Yangoon, Myanmar]; Chittagong [in Bangladesh].

##### Type material.

Lectotype (design. n.), NHMUK 1888.12.4.975 (Fig. 15H; H=30.3 mm, W=16.7 mm), paralectotypes NHMUK 1888.12.4.971–974 (4S, Fig. 15I), NHMUK 1888.12.4.976–979 (4S) from Pegu; SMF 7639 (1S), SMF 175769 (2S) from Chittagong.

##### Remarks.

Fulton clearly stated in the original description that the type series was composed of two lots from Pegu, and Chittagong. No specimens from Chittagong were located in the NHM collections. However, the specimen that most closely matched with the original description in Fulton (1896a: 80) and is figured in Hanley and Theobald (1876: pl. 21, fig. 5) is designated here as the lectotype, NHMUK 1888.12.4.975. The type locality of these taxa is here restricted to “Pegu”, the locality of the lectotype.

#### Amphidromus webbi

Taxon classificationAnimaliaStylommatophoraCamaenidae

Fulton, 1907

Amphidromus webbi Fulton, 1907: 152–153, pl. 9, fig. 8.

##### Type locality.

Nias Island, Sumatra [North Sumatra, Inonesia].

##### Type material.

Holotype NHMUK 1907.5.3.125 (Fig. 15J; H=51.1 mm, W=29.8 mm).

#### Amphidromus zebrinus

Taxon classificationAnimaliaStylommatophoraCamaenidae

(Pfeiffer, 1861)

Bulimus zebrinus Pfeiffer, 1861: 194.

##### Type locality.

Siam [Thailand].

##### Type material.

Lectotype NHMUK 19601439 (Fig. 15K; H=24.7 mm, W=11.6 mm).

### Descriptions Genus *Amphidromus* Albers, 1850

#### Subgenus Syndromus

Taxon classificationAnimaliaStylommatophoraCamaenidae

Pilsbry, 1900

##### Type-species.

*Helix contraria* Müller, 1774 by subsequent designation of Zilch (1960: 623).

#### Amphidromus (Syndromus) globonevilli

Taxon classificationAnimaliaStylommatophoraCamaenidae

Sutcharit & Panha sp. n.

http://zoobank.org/B2747236-D3C2-427E-9FE1-CE1F986CF037

**Figure 16.**
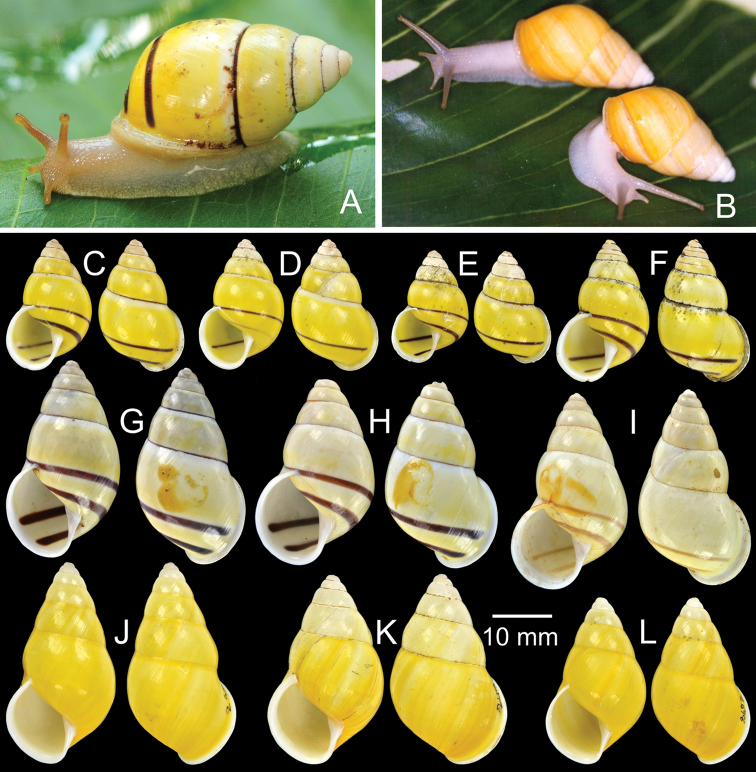
Shells and live snails characteristics. **A** Living snail of Amphidromus (Syndromus) globonevilli Sutcharit and Panha, sp. n. from the type locality with a shell height of approximately 20 mm **B** Living snail of Amphidromus (Syndromus) principalis Sutcharit and Panha, sp. n. from the type locality with a shell height of approximately 25 mm **C–F** Amphidromus (Syndromus) globonevilli Sutcharit and Panha, sp. n. **C** holotype CUMZ 4925 and **D–F** paratypes CUMZ 4927 **G–I** Syntypes UMZC of *Amphidromus sinensis* (Benson, 1851) from China **J–L** Amphidromus (Syndromus) principalis Sutcharit and Panha, sp. n. **J** holotype CUMZ 2543 and **K, L** paratypes CUMZ 2478.

**Figure 17.**
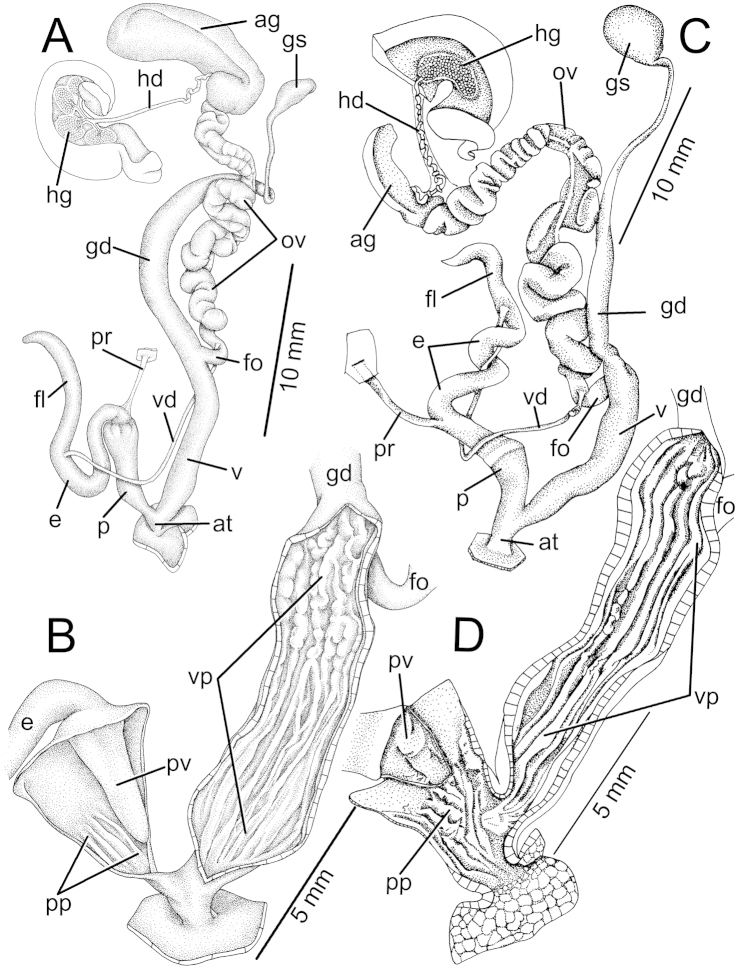
**A–B** Genitalia of Amphidromus (Syndromus) globonevilli Sutcharit and Panha, sp. n. showing the reproductive system and interior structures of the penis and vaginal chamber **C–D** Genitalia of Amphidromus (Syndromus) principalis Sutcharit and Panha, sp. n. showing the general characteristics of the genital system and the interior structures of the penis, atrium and vagina chamber. Anatomical abbreviations are as described in Sutcharit and Panha (2006a, b, 2011): ag, albumin gland; ap, appendix; at, atrium; e, epiphallus; fl, flagellum; fo, free oviduct; gd, gametolytic duct; gs, gametolytic sac; hd, hermaphroditic duct; hg, hermaphroditic gland; o, oviduct; p, penis; pp, penial pilaster; pm, penial retractor muscle; pv, penial verge; v, vagina; vd, vas deferens; vp, vaginal pilaster.

Figs 16A, C–F , 17A, B

Amphidromus sinensis var. globosa Nevill, 1878: 126. [*nomen nudum*]. Type locality: Chittagong [Bangladesh]. Pilsbry 1900: 191. Richardson 1985: 44.

##### Type material.

Holotype CUMZ 4925 (height 21.9 mm, width 14.2 mm, whorls 5¾; Fig. 16C), paratypes CUMZ 4926 (13 shells), CUMZ 4927 (6 shells; Fig. 16D–F), CUMZ 4928 (12 shells), CUMZ 4929 (4 shells), NHMUK 20140707 (2 shells), SMF (2 shells).

Measurement of 37 paratypes; height range 18.3–23.3 mm, mean 21.08 ± 1.18; width range 11.9–15.3 mm, mean 13.59 ± 0.69; height/width ratio 1.46–1.62, mean 1.55 ± 0.04; whorls 5–6.

##### Other material.

Chittagong [now in Bangladesh] original specimen of “*globosa* Nevill, 1878” NHMUK 1903.7.1.1921.

##### Type locality.

Wat Phothikhun, Maesod, Tak, Thailand (16°45'42.2"N, 98°38'49"E).

##### Diagnosis.

This new species can be distinguished from *Amphidromus sinensis* (Benson, 1851) by having a smaller, more ovate conic shell (Fig. 16G–I). It differs from *Amphidromus flavus* (Pfeiffer, 1861) which exhibits an elongated conic shell a faint yellowish spiral band below the periphery, and an elongated aperture (Fig. 7I, J). It differs from *Amphidromus lepidus* (Gould, 1856) and *Amphidromus roemeri* (Pfeiffer, 1863) by having a yellowish shell colour with two dark brown spiral bands below the periphery, while *Amphidromus roemeri* have a more ovate to stout shell, whitish in colour with reddish-brown spiral bands below the periphery (Fig. 13E, F) and *Amphidromus lepidus* has a monochrome whitish shell (Fig. 10C).

##### Description.

**Shell.** Shell small, sinistral, ovate conic, rather thin; umbilicus perforate. Apex acute without black spot; spire short; suture depressed and wide. Whorls slightly convex; last whorl round to ovate. Periostracum thin and transparent. Shell colour yellowish, paler near apex; subsutural band white and with darker yellow band below. Last whorl with two brown spiral bands below periphery. Aperture wide and ovate; columella straight; lip white and little expanded; parietal callus thin and transparent.

##### Genital organs.

Atrium (at) short (n = 5). Penis (p) long, cylindrical and enlarged near penial retractor muscle. Epiphallus (e) smaller than penis and almost similar to penis length; flagellum (fl) similar length to epiphallus; appendix absent. Penial retractor muscle (pr) short and relatively thin. Vas deferens (vd) narrow tube extending from free oviduct (fo) and connected to epiphallus (Fig. 17A).

Internal wall of penis almost smooth surfaced, corrugated into a series of thickened; proximal to genital orifice, with swollen longitudinal penial pilasters (pp). Penial verge (pv) large, elongated conical shape, about two-thirds of penis length and with smooth surface (Fig. 17B).

Vagina (v) cylindrical, longer than penis, held in position with series of thin muscles originating from foot floor. Vaginal pouch and stimulator pilaster absent. Gametolytic duct (gd) long, slender; proximal to genital orifice enlarged same diameter as vagina, and distal to genital orifice tapering to small tube connected to gametolytic sac (gs). Oviduct (ov) and albumen gland (ag) enlarged; hermaphroditic gland (hg) multilobed and connected with hermaphroditic duct (hd) (Fig. 17A).

Internally, vaginal wall sculptured with longitudinal vaginal pilasters (vp); proximal to genital orifice with smooth and continuous ridges about two-third of its length, and pilasters at distal to genital orifice interrupted by transverse divisions (Fig. 17B).

##### Etymology.

The specific name comes from the Latin word “*globous*” meaning “ball or sphere” and the name of Dr. Geoffroy Nevill, who first recognized this as a new species and introduce the name “*globosa*” but was unavailable (see Remark of “*globosa*”).

##### Distribution.

This new species is known from the type locality in Tak Province, western Thailand. In addition, NHM specimens indicate that this species is also found from Chittagong, Bangladesh.

##### Remarks.

The type speceimen of *Amphidromus sinensis* s.s. was presumed to be lost (Pilsbry 1900, Laidlaw and Solem 1961). Recently, we have located one lot of three shells in Benson’s collection at UMZC with the collection locality of “China”, which we consider to be possible syntypes. Photographs of these three shells (Fig. 16G-I) are shown here for further comparison. The shell that most closely matches the original description of Benson’s (1851: 264) and Benson’s specimen figured in Küster and Pfeiffer (1853: pl. 20, figs 1, 2) is illustrated inFigure 16G.

#### Amphidromus (Syndromus) principalis

Taxon classificationAnimaliaStylommatophoraCamaenidae

Sutcharit & Panha sp. n.

http://zoobank.org/27D54FEF-42E2-4F30-B04A-A6DF503FC18F

Figs 16B, J–L , 17C, D

##### Type material.

Holotype: CUMZ 2543 (height 33.9 mm, width 17.9 mm, whorls 6; Fig. 16J), paratypes CUMZ 2478 (3 shells, Fig. 16K, L), CUMZ 2386 (19 shells), CUMZ 2387 (18 shells), NHMUK 20140708 (2 shells) and SMF (2 shells).

Measurement of 27 paratypes; height range 25.2–36.0 mm, mean 30.60 ± 2.38; width range 14.5–18.9 mm, mean 16.43 ± 1.09; height/width ratio 1.69–2.00, mean 1.86 ± 0.07; whorls 5¾–6¾.

##### Other material.

From the type locality CUMZ 2401, 2422.

##### Type locality.

Koh Kra, about 30 km off the east coast of Pak Phanang, Nakhon Srithammarat in the Gulf of Thailand (8°23'55"N, 100°44'2"E).

##### Diagnosis.

This new species is distinguished from *Amphidromus globonevilli* Sutcharit and Panha, sp. n. by having a more ovate to elongated conic shell of entirely uniform bright yellow colour. The reproductive organ lacks a vaginal pouch, the penial verge is small and conical. Living snails have an entirely whitish to creamy body; only older snails are likely to have a pale brown head-foot. Superficially, this new species resembles *Amphidromus flavus* from northern Thailand and Laos. However, this new species exhibits a bright yellow, slightly ovate shell, shorter expanded lip and thickened shell, while *Amphidromus flavus* has a slender, pale yellow shell, wide expanded lip with faint spiral band below periphery (Fig. 7I, J).

##### Description.

**Shell.** Shell ovate to slightly elongate conic, glossy, smooth, sinistral and rimate. Apex obtuse with brown to black spot on the tip. Shell uniform golden yellow (without any bands). Last whorl darker yellow than earlier whorls. Spire conic with slightly depressed suture. Aperture ovate; peristome white, narrowly expanded and not reflected. Columella white, straight and perpendicular. Parietal callus thin and translucent.

##### Genital organ.

Atrium (at) slightly long (n = 10). Penis (p) long, cylindrical and enlarged in middle. Epiphallus (e) longer than penis length; flagellum (fl) shorter than epiphallus; appendix absent. Penial retractor muscle (pr) thickened and relatively long (Fig. 17C). Vas deferens (vd) small tube and connected between epiphallus and free oviduct.

Internal wall of penis corrugated into series of thin and longitudinal penial pilasters (pp), which form a thin fringe around penial verge. Penial verge (pv) short conic, surface with thin irregular furrow (Fig. 17C).

Female reproductive organ similar to former described species but differs in that vagina internal wall possesses swollen and nearly smooth longitudinal vaginal pilaster (Fig. 17C, D).

##### Etymology.

The specific epithet is derived from the Latin “*principalis*” meaning “leader” and refers to Her Royal Highness Princess Maha Chakri Sirindhorn who chaired the Plant Genetic Conservation Project as a Royal Initiation to support biodiversity in Thailand. The malacological survey on Koh Kra in 2000 was part of an expedition supported by this project.

##### Distribution.

This new species is known only from the type locality.

##### Remarks.

*Amphidromus principalis* Sutcharit & Panha, sp. n. is known only from the type locality, the granitic island. The forestation type on the island was dry evergreen forest, the snails were found crawling on the tree leaves, trunks or branches of almost all trees up to 10 m height. We also explored two other satellite islands but found no *Amphidromus* on these islands or any other terrestrial snails other than subulinids.
